# Epigenetic Aging and Racialized, Economic, and Environmental Injustice: NIMHD Social Epigenomics Program

**DOI:** 10.1001/jamanetworkopen.2024.21832

**Published:** 2024-07-29

**Authors:** Nancy Krieger, Christian Testa, Jarvis T. Chen, Nykesha Johnson, Sarah Holmes Watkins, Matthew Suderman, Andrew J. Simpkin, Kate Tilling, Pamela D. Waterman, Brent A. Coull, Immaculata De Vivo, George Davey Smith, Ana V. Diez Roux, Caroline Relton

**Affiliations:** 1 Department of Social and Behavioral Sciences, Harvard T. H. Chan School of Public Health, Boston, Massachusetts; 2 MRC (Medical Research Council) Integrative Epidemiology Unit, Population Health Sciences, Bristol Medical School, University of Bristol, United Kingdom; 3 School of Mathematical and Statistical Sciences, National University of Ireland, Galway; 4 Department of Biostatistics, Harvard T. H. Chan School of Public Health, Boston, Massachusetts; 5 Department of Epidemiology, Harvard T. H. Chan School of Public Health, Boston, Massachusetts; 6 Urban Health Collective and Department of Epidemiology and Biostatistics, Dornsife School of Public Health, Drexel University, Philadelphia, Pennsylvania

## Abstract

**Importance:**

Epigenetic age acceleration is associated with exposure to social and economic adversity and may increase the risk of premature morbidity and mortality. However, no studies have included measures of structural racism, and few have compared estimates within or across the first and second generation of epigenetic clocks.

**Objective:**

To determine whether epigenetic age acceleration is positively associated with exposures to diverse measures of racialized, economic, and environmental injustice measured at different levels and time periods.

**Design, Setting, and Participants:**

This cross-sectional study used data from the My Body My Story (MBMS) study between August 8, 2008, and December 31, 2010, and examination 5 of the Multi-Ethnic Atherosclerosis Study (MESA) from April 1, 2010, to February 29, 2012. In the MBMS, DNA extraction was performed in 2021; linkage of structural measures to the MBMS and MESA, in 2022. US-born individuals were randomly selected from 4 community health centers in Boston, Massachusetts (MBMS), and 4 field sites in Baltimore, Maryland; Forsyth County, North Carolina; New York City, New York; and St Paul, Minnesota (MESA). Data were analyzed from November 13, 2021, to August 31, 2023.

**Main Outcomes and Measures:**

Ten epigenetic clocks (6 first-generation and 4 second-generation), computed using DNA methylation data (DNAm) from blood spots (MBMS) and purified monocytes (MESA).

**Results:**

The US-born study population included 293 MBMS participants (109 men [37.2%], 184 women [62.8%]; mean [SD] age, 49.0 [8.0] years) with 224 Black non-Hispanic and 69 White non-Hispanic participants and 975 MESA participants (492 men [50.5%], 483 women [49.5%]; mean [SD] age, 70.0 [9.3] years) with 229 Black non-Hispanic, 191 Hispanic, and 555 White non-Hispanic participants. Of these, 140 (11.0%) exhibited accelerated aging for all 5 clocks whose estimates are interpretable on the age (years) scale. Among Black non-Hispanic MBMS participants, epigenetic age acceleration was associated with being born in a Jim Crow state by 0.14 (95% CI, 0.003-0.27) SDs and with birth state conservatism by 0.06 (95% CI, 0.01-0.12) SDs, pooling across all clocks. Low parental educational level was associated with epigenetic age acceleration, pooling across all clocks, for both Black non-Hispanic (0.24 [95% CI, 0.08-0.39] SDs) and White non-Hispanic (0.27 [95% CI, 0.03-0.51] SDs) MBMS participants. Adult impoverishment was positively associated with the pooled second-generation clocks among the MESA participants (Black non-Hispanic, 0.06 [95% CI, 0.01-0.12] SDs; Hispanic, 0.07 [95% CI, 0.01-0.14] SDs; White non-Hispanic, 0.05 [95% CI, 0.01-0.08] SDs).

**Conclusions and Relevance:**

The findings of this cross-sectional study of MBMS and MESA participants suggest that epigenetic age acceleration was associated with racialized and economic injustice, potentially contributing to well-documented inequities in premature mortality. Future research should test the hypothesis that epigenetic accelerated aging may be one of the biological mechanisms underlying the well-documented elevated risk of premature morbidity and mortality among social groups subjected to racialized and economic injustice.

## Key Points

**Question:**

Is accelerated epigenetic aging associated with exposure to racialized, economic, and environmental injustice?

**Findings:**

In the US cross-sectional My Body My Story (MBMS) and Multi-Ethnic Atherosclerosis Study (MESA) studies, epigenetic accelerated aging was associated with Jim Crow birth state (MBMS: Black non-Hispanic participants), low parental educational level (MBMS: Black and White non-Hispanic participants), and adult impoverishment (MESA: Black non-Hispanic, Hispanic, and White non-Hispanic participants).

**Meaning:**

These findings suggest that epigenetic accelerated aging may be a biological pathway for embodying racialized and economic injustice.

## Teaser

This cross-sectional study assesses whether socially structured adversity is associated with increased epigenetic accelerated aging among US-born Black non-Hispanic, Hispanic, and White non-Hispanic adults.

## Introduction

Identifying biological pathways by which societal injustice becomes embodied and expressed as population health inequities—that is, unjust, avoidable, and in principle preventable differences in health status across societal groups^1^—is important for both accountability and prevention.^1,2^ Recently, epigenetic pathways have become amenable to investigation in epidemiologic studies^3,4,5^ involving morbidity, clinical treatment, aging, and mortality.^3,4,6^ At issue are diverse biological mechanisms, structured by exposures, canalization, and chance, that regulate gene expression and are mitotically heritable.^3,4,5^

One such mechanism involves DNA methylation (DNAm), which chiefly occurs at cytosine-guanine dinucleotide sites at which a methyl group is added to cytosine and affects gene transcription.^3,4,6^ The mechanisms of DNAm are dynamic and can be transmitted mitotically across cells.^6,7^ In 2013, the first epigenetic “clocks,” using data on age-related temporal patterning of DNAm, were developed to estimate biological age.^7,8,9,10^ Comprising specified sets of DNAm sites, these epigenetic clocks “tick” in all cells and their descendants and yield estimates of DNAm age (or epigenetic age).^7,8^ The first-generation clocks used algorithms trained solely on chronological age; second-generation clocks also use phenotypic training data on mortality, morbidity, and exposures (eg, cigarette smoking) and have also examined the pace of aging within individuals (eTable 1 in Supplement 1).^7,8,11,12^ Epigenetic accelerated aging can thus be quantified as DNAm age exceeding chronological age.^7,8,9,10,11,12^

Since 2020, epidemiologic investigations summarized in several reviews^11,12,13,14^ have reported that epigenetic accelerated aging is associated with increased risk of—and racialized and economic inequities in—cardiometabolic disease, cancer, and earlier age at death. Most commonly studied is individual-level socioeconomic position, with recent evidence (especially for second-generation clocks^11,15,16^) showing stronger associations with early life vs adult deprivation.^11,12,13,14,15,16^ Several studies have reported associations with neighborhood poverty,^11,12,13,14,17^ air pollution,^11,12,13,14,18^ and adult self-reported experiences of racial discrimination^19,20^; none have included measures of structural racism or residential segregation.^21^

Guided by the ecosocial theory of disease distribution and its conceptualization of pathways of embodiment of societal injustice in relation to levels, life course, and historical generation,^2^ we used novel exposure metrics to test the hypothesis that socially structured adversity increases epigenetic accelerated aging. Specifically, we assessed associations between epigenetic accelerated aging among US Black non-Hispanic, Hispanic, and White non-Hispanic adults and exposure to a Jim Crow birthplace (ie, born in a US state in which White supremacy, before the passage of the 1964 and 1965 US Civil Rights Acts, was upheld by both state law and terror^2,22,23,24,25^) and current racialized economic segregation,^25,26,27^ along with exposure to self-reported experiences of racial discrimination, socioeconomic position over the life course, and concurrent air pollution exposure.

## Methods

This cross-sectional study was approved by the Harvard T. H. Chan School of Public Health Office of Human Research; all participants provided written informed consent. The study followed the Strengthening the Reporting of Observational Studies in Epidemiology (STROBE) reporting guideline.

### Study Populations

We used data from 2 preexisting US studies and added new exposure data to both datasets. As prespecified in our study protocol regarding the study setting, the My Body My Story (MBMS) study^28,29^ comprised our discovery dataset (Boston, Massachusetts; 2008-2010), and a subset of the Multi-Ethnic Study of Atherosclerosis (MESA) study^30,31^ served as a secondary dataset (4 US cities; 2010-2010) for data triangulation.^32^

The MBMS study was designed by several current team members (N.K., J.T.C., and P.D.W.) to investigate how racial discrimination and other social exposures affect risk of cardiovascular disease^28,29^; testing such hypotheses requires use of data on racialized group membership.^25,28,29^ As previously reported, the study recruited 1005 participants randomly selected from the patient rosters of 4 Boston community health centers between August 8, 2008, and December 31, 2010, who met study eligibility criteria: adults aged 35 to 64 years (working age), born in the US, who self-reported identifying being Black non-Hispanic or White non-Hispanic.^28,29^ Among the 1005 MBMS participants, 875 (87.1%) provided a finger prick blood sample onto filter paper (409 Black non-Hispanic and 466 White non-Hispanic) and within each racialized group, those who did vs did not provide a blood spot did not differ by income level or self-reported experiences of racial discrimination. Participants’ blood spots were stored at −20 °C and not analyzed until the present investigation.

MESA, a longitudinal cohort, at baseline (2000-2002), recruited 6184 participants free of cardiovascular disease and aged 45 to 84 years who self-reported being African American, Chinese, Hispanic, and White and were recruited from university-affiliated field centers in 6 US communities (Baltimore, Maryland; Forsyth County, North Carolina; New York City, New York; St Paul, Minnesota; Chicago, Illinois; and Los Angeles, California).^30,31^ At examination 5 (April 1, 2010, to February 29, 2012), DNAm was obtained from a randomly selected subset of 1264 participants aged 55 to 94 years (270 Black non-Hispanic, 404 Hispanic, 582 White non-Hispanic, and 10 non-Hispanic of additional racialized groups) drawn from the Baltimore, Forsyth County, New York City, and St Paul recruitment sites.^30,31^ MESA data for examinations 1 to 5 included social metrics analogous to those in MBMS.^30,31^ Since all MBMS participants were born in the US, we restricted analyses to the 975 US-born MESA participants (229 Black non-Hispanic, 191 Hispanic, and 555 White non-Hispanic).

### Epigenetic Data

We extracted DNA from the MBMS blood spots in 2021, using the QIAamp DNA Investigator Kit for FTA and Guthrie cards (QIAGEN), with samples randomized across 96 well plates, and measured DNAm using a commercially available array (Infinium MethylationEPIC Beadchip [Illumina Inc]); protocol details are provided in the preliminary study by Watkins et al^33^ using the MBMS blood spots to demonstrate the feasibility and validity of analyzing specimens with as little as 40 ng of DNA. Among the 875 MBMS blood spot specimens, 472 were judged suitable for DNA extraction; exclusions were due to an initial blood spot protocol problem affecting only the first community health center, whose members were predominantly White non-Hispanic. We then removed specimens from 50 participants with less than 40 ng of DNA and 96 participants with poor-quality DNA extraction^33^ and 35 participants whose self-reported cisgender identity did not match their chromosomal sex as predicted by meffil (an algorithm which relies on staining intensities for the X and Y chromosomes^34^); these latter specimens also displayed very little correlation between chronological age and epigenetic age. In total, 293 MBMS participants (224 Black non-Hispanic and 69 White non-Hispanic) and 857 774 sites passed quality control. Functional normalization included BeadArray (Illumina Inc) as a fixed effect. Blood cell composition was estimated using a deconvolution algorithm,^35^ implemented in meffil, based on the “blood gse35069 complete” cell type reference.

DNAm for the MESA participants was measured in purified primary monocytes obtained from blood drawn in the morning after a 12-hour fast.^36^ As previously described, epigenome-wide methylation was quantified using the Illumina Human Methylation 450 Bead Chip.^36^

We constructed 10 epigenetic clocks using code or formula provided by the developers; values for GrimAge were calculated by uploading data to the GrimAge website (eTable 1 in Supplement 1). Six of these clocks were first generation (Horvath, Hannum, Zhang Age, epiTOC, MiAge, and DNAmTL) and 4 were second generation (Zhang Mortality, PhenoAge, DunedinPoAm, and GrimAge). Shared cytosine-guanine dinucleotide sites were more evident among the first-generation clocks (eTables 1-3 in Supplement 1). To address underlying genetic variability, we computed both surrogate variables and genetic principal components (eAppendix 1 in Supplement 1) and included only the former in our models, noting that inclusion of genetic principal components (available only for MESA) did not alter results (eFigure 1 in Supplement 1). Correlations between chronological and epigenetic age are provided in eFigure 2 in Supplement 1.

### Exposure Data

We conceptualized and operationalized the MBMS and MESA social metric exposure data (details in eAppendix 2 in Supplement 1) in relation to type, level, and period of exposure (at birth, childhood, and adulthood) and stratified analyses by racialized groups, given likely differences in exposure to and impacts of racialized injustice. Measures of exposure to racial injustice at the time of birth included (with the latter two newly added): (1) born in a Jim Crow state^22,25,37^; (2) city of birth Index of Concentration at the Extremes (ICE) for racialized segregation^25,26,27^ (available for MBMS only); and (3) state of birth US state policy liberalism index, drawing on 148 policies enacted over 8 decades, including civil rights legislation.^38^ Validated adult measures of self-reported exposure to racial discrimination included, for MBMS, the Experiences of Discrimination scale,^25,39^ and, for MESA, the Major Discrimination Scale, restricted to unfair treatment attributed to “race”.^25,39^ Self-reported metrics for socioeconomic position included the highest level of educational attainment for the participants and for their parents and the adult socioeconomic data at the individual and household levels for household income, household income per capita, household income to poverty ratio, employment status (and, if employed, occupational class [available in MBMS only]), and housing tenure.^26,28,29^ Based on MBMS and MESA participants’ residential address at the time of the survey, we appended (1) newly generated census tract level data (using the American Community Survey 5-year estimates for 2008-2012^40^) for composition by racialized group and ICE measures for income, racialized, and racialized economic segregation, and housing tenure^25,26,27^; and (2) air pollution data (details provided in Table and eAppendix 2 in Supplement 1). Missingness of the exposure data was typically less than 5% for most variables (Table), and correlations among continuous variables from MBMS and MESA are provided in eFigures 3 and 4 in Supplement 1, respectively. Additional covariates consisted of self-reported data for age, gender, and household size and number of children younger than 18 years (used to determine the household income-to-poverty ratio^41^).

**Table.** Exposure and Covariate Data for US-Born Participants With Epigenetic Data

Variable	Dataset by study population^a^				
	MBMS (n = 293)		MESA, examination 5 (n = 975)		
	Black non-Hispanic (n = 224)	White non-Hispanic (n = 69)	Black non-Hispanic (n = 229)	Hispanic (n = 191)	White non-Hispanic (n = 555)
**Sociodemographic data at time of survey**					
Age, mean (SD), y	49.0 (7.8)	48.7 (8.3)	71.0 (8.9)	68.4 (8.9)	70.1 (9.5)
Missing	0	0	0	0	0
Gender^b^					
Women	135 (60.3)	49 (71.0)	133 (58.1)	86 (45.0)	264 (47.6)
Men	89 (39.7)	20 (29.0)	96 (41.9)	105 (55.0)	291 (52.4)
Missing	0	0	0	0	0
Household size, mean (SD)	3.9 (2.2)	4.0 (1.9)	1.8 (1.1)	2.0 (1.3)	2.0 (1.5)
Missing	1 (0.4)	0	4 (1.7)	3 (1.6)	5 (0.9)
No. of children in household, mean (SD)	1.8 (1.2)	1.9 (1.1)	0.2 (0.6)	0.2 (0.6)	0.1 (0.5)
Missing	1 (0.4)	0	8 (3.5)	8 (4.2)	15 (2.7)
**Childhood exposure to racialized and economic adversity**					
Structural racism: born in a Jim Crow state	71 (31.7)	2 (2.9)	165 (72.1)	19 (9.9)	166 (29.9)
Missing	0	0	0	0	0
Racialized segregation for US city of birth at time of birth, ICE, mean (SD)^c^	0.6 (0.3)	0.8 (0.2)	NA	NA	NA
Missing	13 (5.8)	22 (31.9)	NA	NA	NA
State policy liberalism index for US state of birth at time of birth, mean (SD)^d^	0.7 (1.2)	1.3 (0.5)	−0.1 (1.0)	0.4 (0.7)	0.3 (0.7)
Missing	3 (1.3)	3 (4.3)	2 (0.9)	62 (32.5)	5 (0.9)
Parent highest educational level attained					
<High school	29 (18.4)	8 (14.0)	95 (42.2)	129 (69.7)	161 (29.3)
High school graduate and <4 y college	94 (59.5)	24 (42.1)	106 (47.1)	51 (27.6)	258 (47.0)
≥4 y College	35 (22.2)	25 (43.9)	24 (10.7)	5 (2.7)	130 (23.7)
Missing	66 (29.5)	12 (17.4)	4 (1.7)	6 (3.1)	6 (1.1)
Participant highest educational level attained					
<High school	34 (15.2)	8 (11.6)	23 (10.0)	35 (18.3)	21 (3.8)
High school graduate and <4 y college	161 (71.9)	33 (47.8)	175 (76.4)	140 (73.3)	413 (74.4)
≥4 y College	29 (12.9)	28 (40.6)	31 (13.5)	16 (8.4)	121 (21.8)
Missing	0	0	0	0	0
**Adult self-reported exposure to racial discrimination** ^e^					
EOD scale, No. of domains					
0	30 (13.6)	35 (50.7)	NA	NA	NA
1-2	52 (23.4)	24 (34.8)	NA	NA	NA
≥3	140 (63.1)	10 (14.5)	NA	NA	NA
Missing	2 (0.9)	0	NA	NA	NA
MDS self-reported exposure to unfair treatment with main reason attributed to race or ethnicity, No. of domains					
0	NA	NA	129 (56.6)	131 (68.6)	534 (96.4)
1-2	NA	NA	79 (34.6)	53 (27.7)	20 (3.6)
≥3	NA	NA	20 (8.8)	7 (3.7)	0
Missing	NA	NA	1 (0.4)	0	1 (0.2)
**Adult household socioeconomic position**					
Household income, mean (SD), 2010 US$	43 900.2 (41 430.5)	60 008.0 (45 750.5)	52 345.4 (35 133.9)	47 035.8 (30 778.9)	64 743.3 (39 209.9)
Missing	33 (14.7)	3 (4.3)	8 (3.5)	5 (2.6)	(23 (4.1)
Household income, mean (SD), 2010 US$ per capita	13 534.5 (15 444.8)	17 430.2 (15 353.2)	31 561.9 (19 550.8)	26 956.5 (19 484.4)	38 073.3 (25 989.4)
Missing	34 (15.2)	3 (4.3)	9 (3.9)	7 (3.7)	24 (4.3)
Household income-to-poverty ratio level, mean (SD)	2.2 (2.2)	2.9 (2.3)	3.9 (2.3)	3.3 (2.1)	4.8 (2.9)
Missing	34 (15.2)	3 (4.3)	9 (3.9)	7 (3.7)	24 (4.3)
Household below US poverty line	89 (46.8)	17 (25.8)	11 (5.0)	13 (7.1)	17 (3.2)
Missing	34 (15.2)	3 (4.3)	9 (3.9)	7 (3.7)	24 (4.3)
Occupational class of participant					
Employed	119 (53.1)	42 (60.9)	79 (34.5)	89 (46.6)	267 (48.1)
Nonsupervisory employee	76 (33.9)	16 (23.2)	NA	NA	NA
Owner, self-employed, or supervisory employee	43 (19.2)	26 (37.7)	NA	NA	NA
Missing	0	0	NA	NA	NA
Unemployed or not in the paid labor force	105 (46.9)	27 (39.1)	150 (65.5)	102 (53.4)	288 (51.9)
Missing	0	0	0	0	0
Housing tenure					
Home owned with a mortgage/loan	40 (20.6)	32 (50.0)	90 (40.9)	77 (42.3)	266 (48.6)
Home owned free and clear	7 (3.6)	6 (9.4)	44 (20.0)	29 (15.9)	211 (38.6)
Rent home	147 (75.8)	26 (40.6)	86 (39.1)	76 (41.8)	70 (12.8)
Missing	30 (13.4)	5 (7.2)	9 (3.9)	9 (4.7)	8 (1.4)
**Exposure to air pollution**					
Annual black carbon exposure, mean (SD), μg/m^3^^f^	0.6 (0.1)	0.6 (0.2)	NA	NA	NA
Missing	0	0	NA	NA	NA
Light absorption coefficient for black carbon exposure estimate, mean (SD)^g^	NA	NA	0.9 (0.3)	0.7 (0.4)	0.6 (0.3)
Missing	NA	NA	14 (6.1)	11 (5.8)	23 (4.1)
Pollution Proximity Index, mean (SD)^h^	4.3 (1.1)	3.9 (1.4)	NA	NA	NA
Missing	5 (2.2)	0	NA	NA	NA
Nitrous oxides, mean (SD), parts per billion	NA	NA	31.9 (16.2)	27.0 (16.4)	21.5 (12.2)
Missing	NA	NA	4 (1.7)	11 (5.8)	23 (4.1)
**Census tract characteristics, ACS 5-y estimate (2008-2012)**					
Composition by racialized group, mean (SD), %					
American Indian or Alaska Native	0.2 (0.6)	0.1 (0.3)	0.2 (0.6)	0.3 (0.6)	0.3 (0.5)
Asian non-Hispanic	5.9 (7.0)	9.5 (10.8)	2.4 (3.5)	5.8 (6.7)	5.3 (4.9)
Black non-Hispanic	43.9 (27.6)	14.8 (16.7)	62.4 (31.4)	13.8 (13.9)	16.9 (19.3)
Hispanic	19.2 (11.6)	14.5 (11.7)	15.3 (21.3)	26.9 (23.7)	14.4 (14.2)
Native Hawaiian and Other Pacific Islander	0 (0.1)	0 (0)	0 (0.3)	0 (0.1)	0 (0.2)
White non-Hispanic	26.9 (26.5)	58.3 (26.4)	17.6 (23.0)	50.5 (31.3)	60.7 (23.8)
Missing	0	0	2 (0.9)	12 (6.3)	2 (0.4)
ICE^i^					
Income, mean (SD)	−0.12 (0.25)	0.10 (0.29)	−0.08 (0.25)	−0.02 (0.24)	0.09 (0.23)
Missing	0	0	2 (0.9)	12 (6.3)	4 (0.7)
Racial segregation, mean (SD)^j^	−0.17 (0.53)	0.43 (0.41)	−0.45 (0.50)	0.37 (0.42)	0.44 (0.40)
Missing	0	0	2 (0.9)	12 (6.3)	2 (0.4)
Racialized economic segregation, mean (SD)^k^	−0.07 (0.22)	0.19 (0.20)	−0.11 (0.21)	0.09 (0.18)	0.16 (0.18)
Missing	0	0	2 (0.9)	(12 (6.3)	(4 (0.7)
Housing tenure, mean (SD)^l^	−0.34 (0.42)	−0.10 (0.44)	−0.07 (0.59)	−0.04 (0.58)	0.22 (0.41)
Missing	0	0	2 (0.9)	12 (6.3)	4 (0.7)
Current smokers	115 (51.3)	24 (34.8)	31 (13.6)	16 (8.5)	44 (8.0)
Missing	0	0	1 (0.4)	2 (1.0)	4 (0.7)
BMI	32.11 (7.74)	29.71 (7.24)	30.58 (5.68)	30.81 (5.49)	28.70 (5.35)
Missing	0	0	0	0	1 (0.2)

Abbreviations: ACS, American Community Survey; BMI, body mass index (calculated as the weight in kilograms divided by the height in meters squared); EOD, Experiences of Discrimination; ICE, Index of Concentration at the Extremes; MBMS, My Body My Story; MDS, Major Discrimination Scale; MESA, Multi-Ethnic Atherosclerosis Study; NA, not applicable.

^a^ Unless otherwise indicated, data are expressed as No. (%) of participants. Percentages have been rounded and may not total 100. In those cases where the No. (%) missing exceed 0, the values presented for the continuous (mean [SD]) and categorical (No. [%]) data are based on the observed data.

^b^ We use the sex/gender terminology employed in each study in the self-report questions asked of each participant, and do not have data as to whether participants identified as cisgender, transgender, or non-binary/gender diverse.

^c^ Range from −1 (100% Black) to 1 (100% White) among US-born participants.

^d^ Range from −2.5 (least liberal) to 2.8 (most liberal).

^e^ The EOD scale ranges from 0 to 9 domains of self-reported exposure to racial discrimination; the MDS ranges from 0 to 6 domains of self-reported unfair treatment due to race.

^f^ For MBMS only: model estimates of average annual black carbon in the atmosphere at the participants' resident address for the year prior to their survey data.

^g^ For MESA only: model estimated average for 2-week predictions from January 2000 to December 2010, generated at each participant’s baseline average; a light absorption coefficient of 0.5 × 10^−5^ m is approximately equal to 0.5 μm^3^ of black carbon.

^h^ For MBMS only: the Pollution Proximity Scores (0-5) is based on the quintile ranges of emissions intensity values in 2012 across 6 pollutants (carbon monoxide, sulfur dioxide, nitrogen dioxide, nitrogen oxide, particulate matter less than 2.5 μm in diameter, and carbon dioxide).

^i^ ICE for economic segregation extremes set as (1) high-income households (top quintile of US household income) and (b) low-income households (bottom quintile of US household income), at time of survey. Range: −1 (100% low-income households) to 1 (100% high-income households).

^j^ ICE for racialized segregation extremes set as (1) Black non-Hispanic persons and (2) White non-Hispanic persons, at time of survey. Range: −1 (100% Black non-Hispanic individuals) to 1 (100% White non-Hispanic individuals).

^k^ ICE for racialized economic segregation extremes set as (1) Black low-income non-Hispanic households and (2) White high-income non-Hispanic households, at time of survey. Range: −1 (100% Black low-income non-Hispanic households) to 1 (100% White high-income non-Hispanic households).

^l^ ICE for housing tenure segregation extremes set as (1) homeowner households and (2) renter households, at time of survey. Range: −1 (100% renter households) to 1 (100% homeowner households).

### Statistical Analysis 

Data were analyzed from November 13, 2021, to August 31, 2023. Epigenetic clocks were regressed jointly on participants’ age, exposures to adversity (Table), sex and/or gender, body mass index (BMI; calculated as the weight in kilograms divided by the height in meters squared), smoking, cell type proportions in their blood sample, and surrogate variables (eAppendix 1 in Supplement 1). Using R (with R packages listed in eTable 2 in Supplement 1 [R Project for Statistical Computing]), we fit separate models for each exposure-clock combination. Models were standardized to account for clocks’ scale differences, with effect estimates presented as differences (in units of SDs for each clock) for a 1-unit change in predictive factors. We used crossed random-effects models^42^ with random intercepts by participant and random age slopes by clock to estimate mean exposure effects assuming a common latent epigenetic aging construct observable across clocks. We used multiple imputation to account for missing data (eAppendix 3 in Supplement 1) and Bonferroni and false discovery rate (FDR) correction for multiple comparisons testing and set the overall significance level (2-sided) .05. Results were only considered significant if the *P* value of the model estimates were less than the calculated *P* value threshold, which varied based on the number of comparisons (eAppendix 3 in Supplement 1).

## Results

The Table summarizes the distribution of the exposure data and covariates for the 293 US-born MBMS study participants and the 975 US-born MESA study participants with DNAm data, overall and stratified by racialized group (MBMS, 224 Black non-Hispanic and 69 White non-Hispanic; MESA, 229 Black non-Hispanic, 191 Hispanic, and 565 White non-Hispanic). Participants in MBMS were younger than MESA participants (mean [SD], 49.0 [8.0] vs 70.0 [9.3] years) and more likely to self-identify as being women (184 [62.8%] and 109 men [37.2%] compared with 483 women [49.5%] and 492 men [50.5%]). As expected, the Black non-Hispanic participants in both MBMS and MESA were most likely to be born in a Jim Crow state and/or a state with a low (least liberal) state policy liberalism index, and, as adults, to self-report exposure to racial discrimination in 3 or more domains and reside in segregated census tracts with high extreme concentrations of Black and low-income Black non-Hispanic households (Table). By contrast, the US-born White non-Hispanic MBMS and MESA participants were most likely to live in census tracts with high extreme concentrations of, respectively, White and high-income non-Hispanic White households (Table). Educational attainment was lower for participants’ parents compared with the participants, and in both groups was lowest for the Black non-Hispanic and Hispanic participants (Table). Similar patterns occurred for self-reported adult household income, poverty level, occupational class (if employed), and housing tenure and for the ICE metrics for income segregation and housing tenure.

In eTable 4 in Supplement 1, we present, for each epigenetic clock, the MBMS and MESA DNAm raw age estimate and epigenetic accelerated aging estimates (detrended for chronological age) in relation to each specified exposure, stratified by racialized group, and the cell type proportions. Among all study participants, 140 (11.0%) exhibited accelerated aging for all 5 clocks whose estimates are interpretable on the age (years) scale (eTable 4 in Supplement 1).

**Figure 1.**
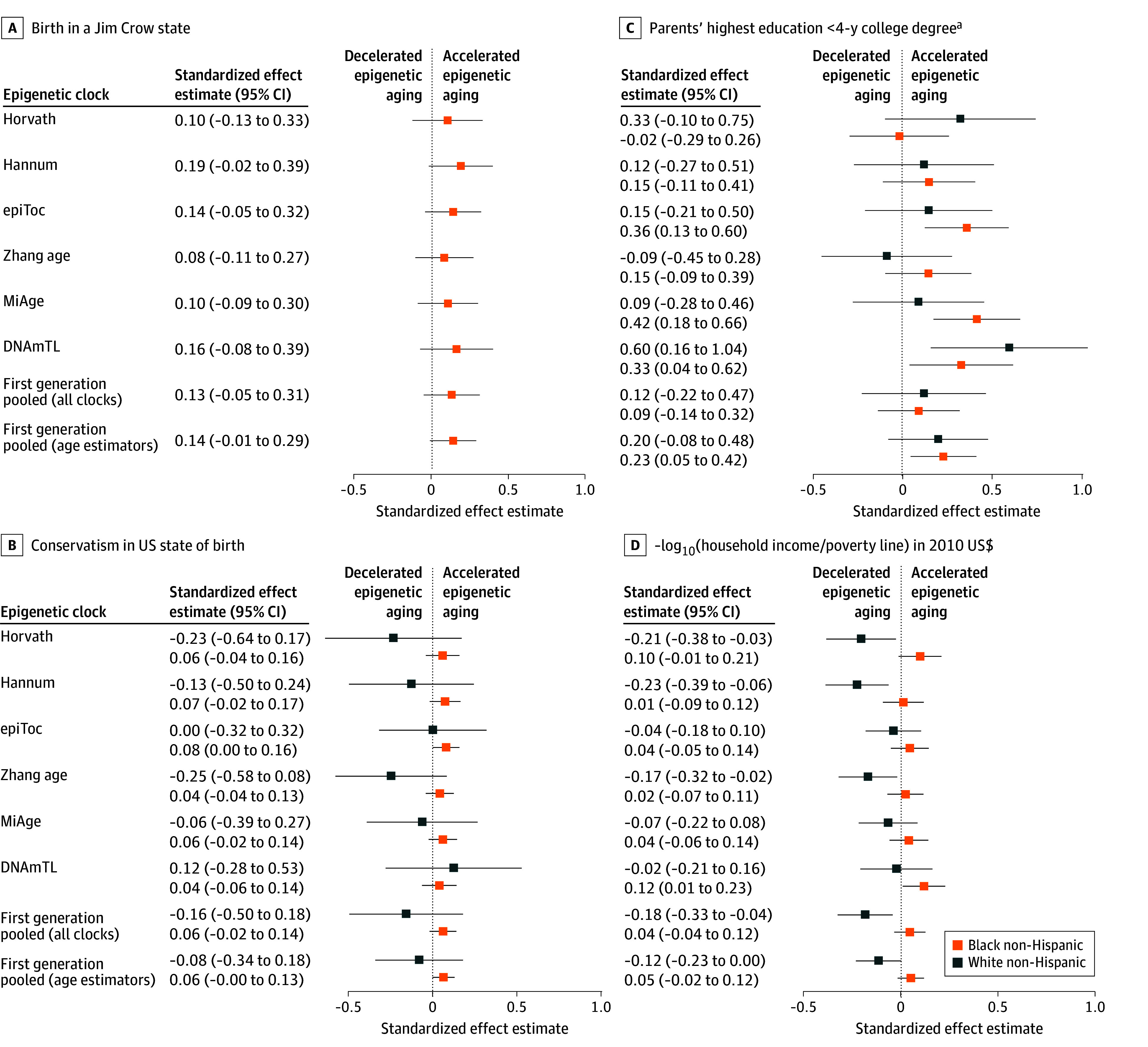
Standardized Effect Estimates (Unadjusted) for Early Life Racialized and Economic Adverse Exposures and Adult Impoverishment Among My Body My Story Study Participants: First-Generation Epigenetic Clocks Whiskers indicate 95% CIs. ^a^Reference: parents with a 4-year college degree.

**Figure 2.**
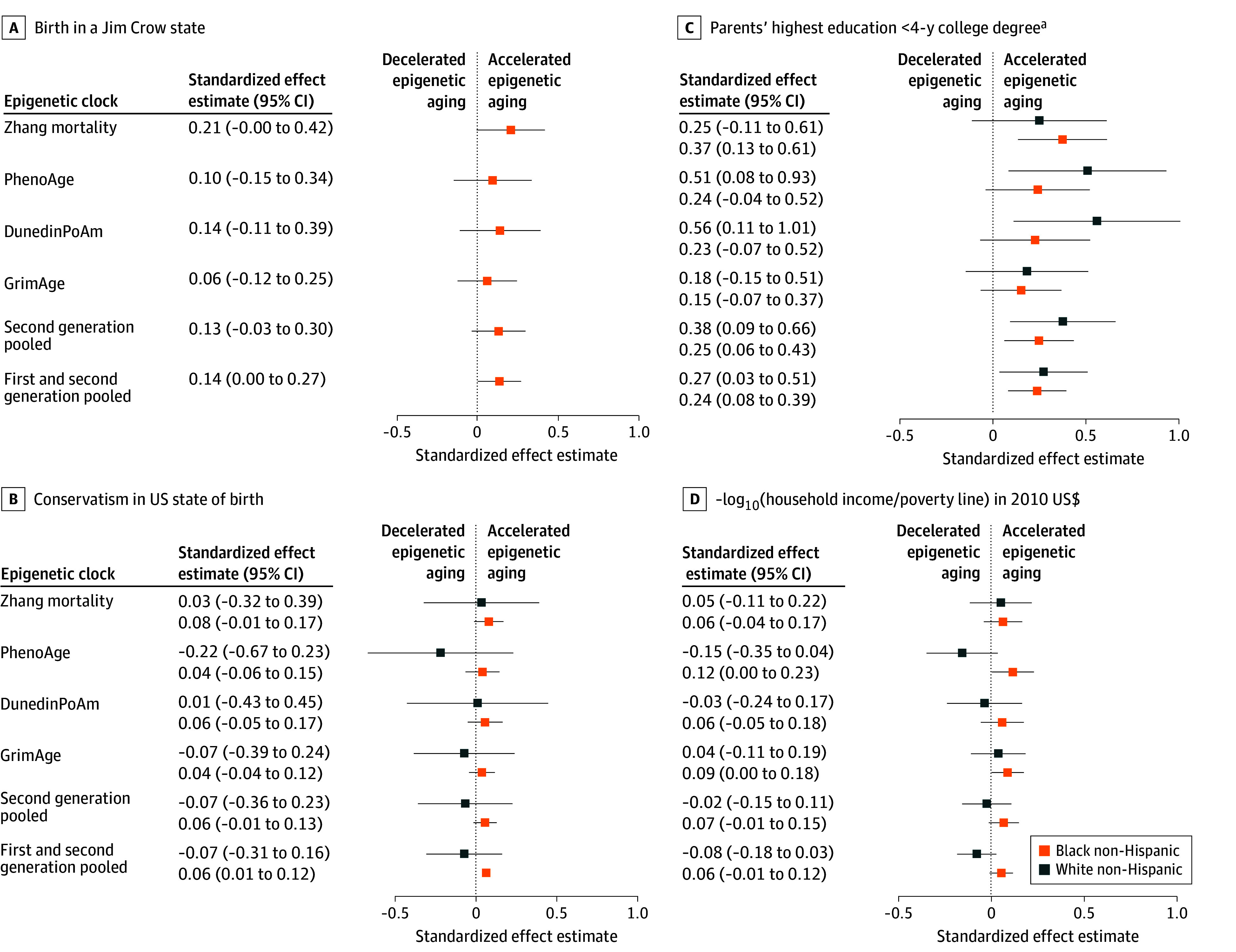
Standardized Effect Estimates (Unadjusted) for Early Life Racialized and Economic Adverse Exposures and Adult Impoverishment Among My Body My Story Study Participants: Second-Generation Epigenetic Clocks Whiskers indicate 95% CIs. ^a^Reference: parents with a 4-year college degree.

**Figure 3.**
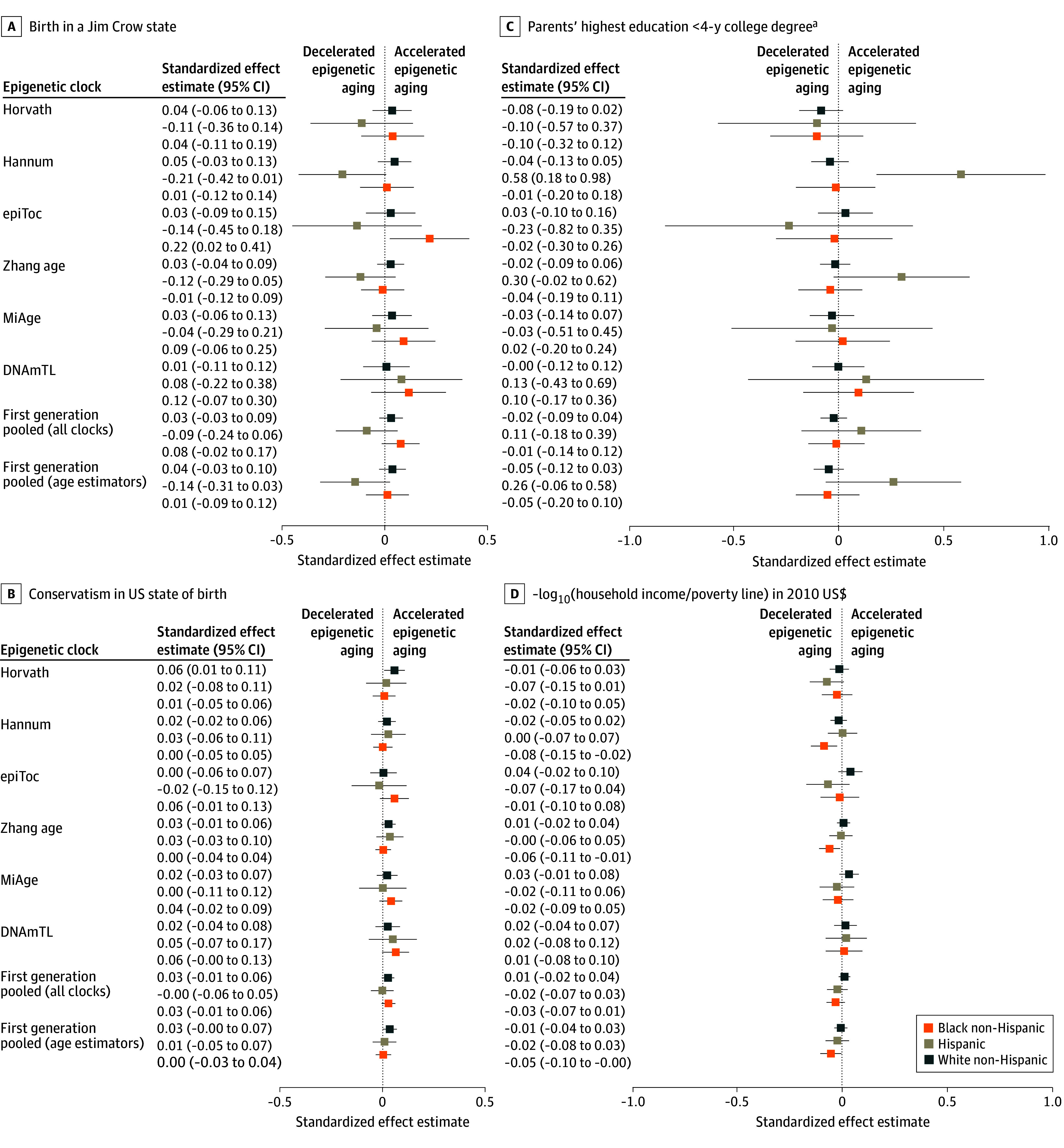
Standardized Effect Estimates (Unadjusted) for Select Early Life Racialized and Economic Adverse Exposures and Adult Impoverishment Among Multi-Ethnic Atherosclerosis Study Participants: First-Generation Epigenetic Clocks Whiskers indicate 95% CIs. ^a^Reference: parents with a 4-year college degree.

**Figure 4.**
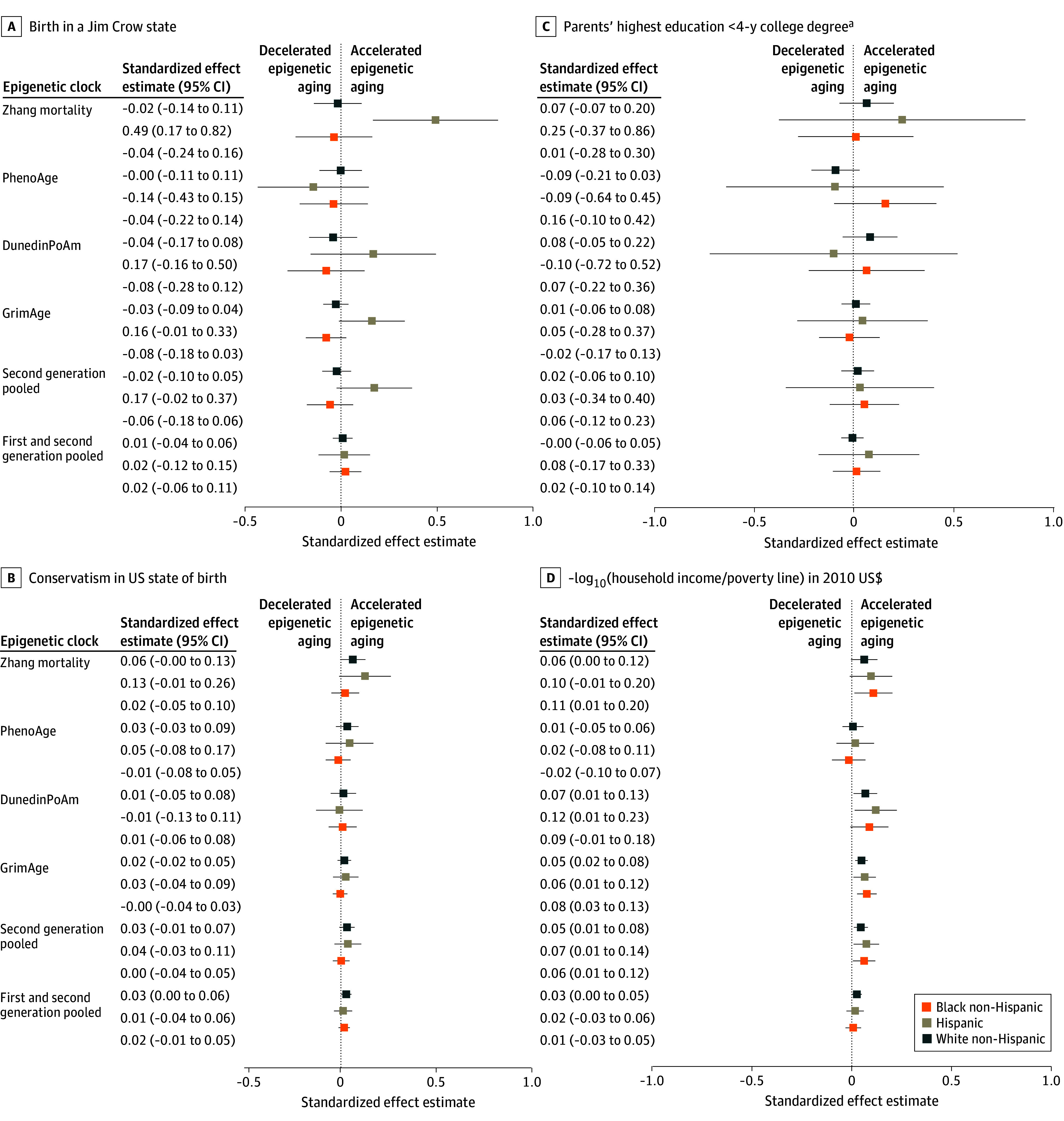
Standardized Effect Estimates (Unadjusted) for Select Early Life Racialized and Economic Adverse Exposures and Adult Impoverishment Among Multi-Ethnic Atherosclerosis Study Participants: Second-Generation Epigenetic Clocks Whiskers indicate 95% CIs. ^a^Reference: parents with a 4-year college degree.

We present standardized effect estimates (unadjusted for multiple comparisons) for each clock and for the pooled clock data, controlling for age, sex or gender, cell-type proportions, surrogate variables, smoking, and BMI, in Figure 1 and Figure 2 (for MBMS participants) and in Figure 3 and Figure 4 (for MESA participants), which include select exposure metrics; eFigures 5 to 10 in Supplement 1 include all exposure metrics. In eFigures 11 to 16 in Supplement 1, we remove smoking and BMI. For interpretation, the effects of the first-generation age-estimator clocks can be viewed as how many additional years of aging (in units of SDs) are associated with the exposures, either compared with the reference category for categorical variables, or according to a 1-unit SD increase in the exposure for the continuous measures. For reference, in the MBMS population, age had an SD of 7.92, so a pooled effect estimate of 0.10 on the age-estimator clocks is comparable to an increase in aging of 0.79 years. Other clocks (as described in eTable 1 in Supplement 1) estimate age-related measures such as mortality risk, mitotic divisions of stem cells, phenotypic outcomes, and telomere length, and therefore cannot be interpreted in units of additional years of aging. To ensure that the common effects of adverse exposures across clocks would be in the same direction, we reversed the direction of the DNAm clock for telomere length (multiplied by −1), because telomere length decreases with age, and reversed the ICE measures compared with their conventional definitions, so that higher values represented more adverse exposures. All models controlled for the specified covariates (age, sex or gender, cell-type proportions, smoking, BMI, and surrogate variables). The Experiences of Discrimination measure refers specifically to those reported by participants as being due to their “race, ethnicity, or color.” Because only 2 of the MBMS White non-Hispanic participants were born in a Jim Crow state (Table), we do not report results for this group, given small numbers. Effect estimates for Figures 1 to 4 are not adjusted for multiple comparisons.

Among MBMS participants (Figures 1 and 2), we detected consistent patterns of epigenetic accelerated aging (with estimates whose 95% CI excluded 0), pooling across all clocks, for 3 social exposures: Jim Crow birthplace (0.14 [95% CI, 0.003-0.27]) SDs and birth state conservatism (0.06 [95% CI, 0.01-0.12]) SDs for Black non-Hispanic participants only and low parental educational level for both Black non-Hispanic (0.24 [95% CI, 0.08-0.39] SDs) and White non-Hispanic (0.27 [95% CI, 0.03-0.51] SDs) participants; these latter estimates were similar or higher for the pooled second-generation clocks for the Black non-Hispanic (0.25 [95% CI, 0.06-0.43] SDs) and the White non-Hispanic (0.38 [95% CI, 0.09-0.66] SDs) participants.

Among the US-born MESA participants, pooling across all clocks (Figures 3 and 4), we observed consistent patterns of accelerated epigenetic aging for the social exposures only for the White non-Hispanic participants for higher adult impoverishment (0.03 [95% CI, 0.001-0.05] SDs) and for birth state conservatism (0.03 [95% CI, 0.01-0.06] SDs). Adult impoverishment was also associated with epigenetic accelerated aging for the pooled second-generation clocks for all 3 racialized groups (Black non-Hispanic, 0.06 [95% CI, 0.01-0.12] SDs; Hispanic, 0.07 [95% CI, 0.01-0.14] SDs; White non-Hispanic, 0.05 [95% CI, 0.01-0.08] SDs).

In the MESA population, age had an SD of 9.36, so a pooled effect estimate of 0.10 on the age-estimator clocks is comparable to an increase in aging of 0.94 years. For the Major Discrimination Score (referring to discrimination reported by participants who attributed their reported unfair experiences as due to their “race”), no White non-Hispanic MESA participants had scores of 3 or greater (Table), so there are no data to report for this category.

Bonferroni correction for multiple comparisons testing increased the *P* values such that no associations were observed with individual clocks. At an FDR below 5%, associations in MBMS were observed for parental education (<4 years of college vs ≥4 years of college) and first- and second-generation clocks pooled for both Black non-Hispanic and White non-Hispanic individuals. No associations at an FDR of less than 0.05 were observed for pooled clocks in MESA. Additional analyses showed the expected associations of epigenetic accelerated aging with chronological age, BMI, and smoking (eTable 5 in Supplement 1) and also little difference in associations for exposures if analyses excluded smoking and BMI (eFigures 11-16 in Supplement 1).

## Discussion

Our cross-sectional study offers intriguing novel evidence that exposure to Jim Crow at the time of birth may be associated with epigenetic accelerated aging (pooling across all clocks) among US-born Black non-Hispanic working age adults, for whom being born in a Jim Crow state translated to age acceleration by 0.14 (95% CI, 0.003-0.27) SDs in MBMS, with evidence also of associations with birth state conservatism. The study also adds to extant evidence that epigenetic accelerated aging, among working age or older adults, is associated with low parental educational level and adult impoverishment.^11,12,13,14,15,16^

Supporting our view that the Jim Crow birthplace and parental education exposures provide suggestive signals of association, new research using second-generation clocks has reported epigenetic accelerated aging to be associated with the economic shock of the 1930s US Great Depression^43^ and parents’ educational attainment.^16,44,45^ Inconsistencies in the MBMS and MESA results for Jim Crow place of birth merit further investigation and plausibly may reflect geographic variability in Jim Crow exposures, Jim Crow migration patterns, and exposure to racism in non–Jim Crow states.^22,23,24,25,37,46^ Other inconsistencies in the MBMS and MESA results may reflect differences in age of participants, cell types used for DNA extraction, and measurement on different Illumina BeadChips (450K vs EPIC). Our results are in accord with studies linking epigenetic accelerated aging to adult economic deprivation and exposure to air pollution,^11,12,13,14,15,16,18,44,45,47^ with evidence indicating adults’ methylome change in response to acute short-term exposures to air pollutants.^48^

### Strengths and Limitations

Study strengths include our parallel analyses across 2 similar but not identical study populations, enhancing robustness of hypothesis testing.^32^ Under the assumption that the different clocks (variously trained on age, mortality, or phenotype data) reflect a latent construct of epigenetic aging, we computed standardized effect estimates to aid comparison of associations, singly and pooled. We also modeled age in our analysis using methods consistent with prior work on identifying the correct way to model age in analyses of accelerated epigenetic aging.^21^

Several study limitations also merit consideration. First, despite having sample sizes on par with or larger than the handful of other epigenetic clock studies with data on exposure to racial injustice,^21^ our analytic sample sizes were relatively small, especially compared with much larger studies with more statistical power (with primarily European or US White populations) that have analyzed epigenetic clocks in relation to economic and pollution exposures.^12,13,14,18,49^ Although a previous investigation^33^ demonstrated that data meeting rigorous quality control protocols using as little as 40 ng of extracted DNA could be obtained, statistical power decreases with decreasing total weight of DNA. We accordingly focus on consistency of point estimates rather than statistical significance or corrections for multiple comparisons.

Second, MBMS used frozen blood spots, a source validated for DNAm analyses,^50^ whereas MESA DNAm was obtained from purified monocytes; we accounted for cell-type proportion in our analyses. Although DNAm patterns can vary by tissue, studies using DNAm measured in blood, including longitudinal analyses, have found robust associations between epigenetic accelerated aging and diverse health outcomes.^11,12,13^ Third, we relied on self-reported data for exposure to racial discrimination, with likely misclassification leading to conservative estimates.^25,28,29^ Fourth, the study was not designed to yield causal effect estimates; generating data on associations with novel exposures, however, is a necessary first step. Foci for our next studies include epigenome-wide association study analyses and investigating whether specific DNAm sites or epigenetic clocks modify associations between the study exposures and selected health outcomes.

## Conclusions

The findings of this cross-sectional study, in conjunction with extant literature, support strengthening research to test the hypothesis that epigenetic accelerated aging may be one of the biological mechanisms underlying the well-documented elevated risk of premature morbidity and mortality, including for noncommunicable diseases, among social groups subjected to, vs protected from, racialized and economic injustice.^2,11,12,13,14,15,16^ Future studies may benefit from inclusion of historically informed measures of structural racialized and economic injustice contingent on the time period and geographic location where people are born, live, work, and bear and birth their children.

## Notes

Supplement 1. **eTable 1.** Primary Citations for and Descriptions of the 10 Epigenetic Clocks Used in This Study **eTable 2.** Software Dependency Citations (Using Citations Specified by Each Source) **eTable 3.** Number of Overlapping CpG Sites Shared Between Clocks **eTable 4.** Chronological Age, Epigenetic Clock and Accelerated Aging Data and Epigenetic Assay Covariates: My Body My Story Study (MBMS; Boston, MA, 2008-2010; Ages 35-64 Years) and Multi-Ethnic Study of Atherosclerosis (MESA; 6 US Sites, Exam 5 Epigenetic Subsample, 2010-2012; Ages 55-94 Years, US-Born) **eTable 5.** Standardized Estimates (Unadjusted) of Epigenetic Age Acceleration Associated With Smoking, Age, and Body Mass Index (BMI), for MBMS and MESA US-Born, Estimate and 95% CIs **eAppendix 1.** Construction of Surrogate Variables and Genetic Principal Component (PC) Variables **eAppendix 2.** Definitions and Sources of Metrics Included in Table 1 **eAppendix 3.** Methods for Multiple Imputation and Multiple Comparisons **eFigure 1.** Additional Variance Explained by Incorporating Genetic PCs **eFigure 2.** Correlation of Chronological Age and Epigenetic Aging by Epigenetic Clock: MESA and MBMS **eFigure 3.** Correlation Matrix for Continuous Variables From MBMS **eFigure 4.** Correlation Matrix for Continuous Variables From MESA, US-Born **eFigure 5.** MBMS: Standardized Effect Estimates (Unadjusted) (95% CI) for Early Life Adverse Exposure and Adult Racial Discrimination **eFigure 6.** MBMS: Standardized Effect Estimates (Unadjusted) (95% CI) for Adult Economic, Pollution, and Segregation Adverse Exposures **eFigure 7.** MESA: Standardized Effect Estimates (Unadjusted) (95% CI) for Early Life Adverse Exposure and Adult Racial Discrimination **eFigure 8.** MESA: Standardized Effect Estimates (Unadjusted) (95% CI) for Adult Economic, Pollution, and Segregation Adverse Exposures **eFigure 9.** Standardized Effect Estimates (Unadjusted) and 95% CIs by Racialized Group for Early Life Adverse Exposures (Reversed Index of Concentration at the Extremes for Racialized Segregation at City of Birth) and Adult Individual-, Household-, and Area-Based Adverse Exposures (Employment Status; Housing Tenure, and Reversed Index of Concentration at the Extremes for Income and Housing Tenure): MBMS Participants **eFigure 10.** Standardized Effect Estimates (Unadjusted) and 95% CIs by Racialized Group for Early Life Adverse Exposures (Reversed Index of Concentration at the Extremes for Racialized Segregation at City of Birth) and Adult Household- and Area-Based Adverse Exposures (Employment Status; Housing Tenure, and Reversed Index of Concentration at the Extremes for Income and Housing Tenure): MESA Participants **eFigure 11.** Standardized Effect Estimates (Unadjusted) by Racialized Group and 95% CIs for Early Life Exposures (Born in a Jim Crow State, Reversed Index of Concentration at the Extremes for Racialized Segregation at City of Birth, State Policy Conservatism in State of Birth, Parents' Education, Participants’ Education): MBMS, Not Including Covariate Data on BMI and Smoking **eFigure 12.** Standardized Effect Estimates (Unadjusted) by Racialized Group and 95% CIs for Adult Life Exposures (Set 1: Experiences of Discrimination, Negative log Household Income Ratio to the Poverty Line, Occupational Class, Employment Status, Housing Tenure, and Black Carbon Air Pollution): MBMS, Not Including Covariate Data on BMI and Smoking **eFigure 13.** Standardized Effect Estimates (Unadjusted) by Racialized Group and 95% CIs for Adult Life Exposures (Set 2: Pollution Proximity Index [Air Pollution], Reversed Index of Concentration at the Extremes for Income, Racial Segregation, Racialized Economic Segregation, and Housing Tenure): MBMS, Not Including Covariate Data on BMI and Smoking **eFigure 14.** Standardized Effect Estimates (Unadjusted) by Racialized Group and 95% CIs for Early Life Exposures (Born in a Jim Crow State, State Policy Conservatism in State of Birth, Parents' Education, Participants’ Education): MESA US-Born, Not Including Covariate Data on BMI and Smoking **eFigure 15**. Standardized Effect Estimates (Unadjusted) by Racialized Group and 95% CIs for Adult Life Exposures (Set 1: Major Discrimination Scale (racialized), Negative log Household Income Ratio to the Poverty Line, Employment Status, Housing Tenure, and Light Absorption Coefficient [Air Pollution]): MESA US-Born, Not Including Covariate Data on BMI and Smoking **eFigure 16.** Standardized Effect Estimates (Unadjusted) by Racialized Group and 95% CIs for Adult Life Exposures (Set 2: Oxides of Nitrogen [Air Pollution], Reversed Index of Concentration at the Extremes for: Income, Racial Segregation, Racialized Economic Segregation, and Housing Tenure): MESA US-Born, Not Including Covariate Data on BMI and Smoking

Supplement 2.
Data Sharing Statement
