# Errors in Figure Labels

**DOI:** 10.1001/jamanetworkopen.2024.35266

**Published:** 2024-08-27

In the Original Investigation titled “Epigenetic Aging and Racialized, Economic, and Environmental Injustice: NIMHD Social Epigenomics Program,”^1^ published July 29, 2024, there were errors introduced in the production process in the x-axis labels and data column headings in Figures 1 through 4. Instead of “OR (95% CI),” the x-axis labels should have been “Standardized effect estimate” and the data column headings should have been “Standardized effect estimate (95% CI).” In addition, the first sentence of all figure captions should be replaced with “Whiskers indicate 95% CIs.” This article has been corrected.^1^
