# Supplementary material for: Epigenetic Aging and Racialized, Economic, and Environmental Injustice: NIMHD Social Epigenomics Program
Source: JAMA Netw Open. 2024 Jul 29;7(7):e2421832. doi: 10.1001/jamanetworkopen.2024.21832 (PMC11287398; doi:10.1001/jamanetworkopen.2024.21832)
Supplement: Supplement 1. — eTable 1. Primary Citations for and Descriptions of the 10 Epigenetic Clocks Used in This Study eTable 2. Software Dependency Citations (Using Citations Specified by Each Source) eTable 3. Number of Overlapping CpG Sites Shared Between Clocks eTable 4. Chronological Age, Epigenetic Clock and Accelerated Aging Data and Epigenetic Assay Covariates: My Body My Story Study (MBMS; Boston, MA, 2008-2010; Ages 35-64 Years) and Multi-Ethnic Study of Atherosclerosis (MESA; 6 US Sites, Exam 5 Epigenetic Subsample, 2010-2012; Ages 55-94 Years, US-Born) eTable 5. Standardized Estimates (Unadjusted) of Epigenetic Age Acceleration Associated With Smoking, Age, and Body Mass Index (BMI), for MBMS and MESA US-Born, Estimate and 95% CIs eAppendix 1. Construction of Surrogate Variables and Genetic Principal Component (PC) Variables eAppendix 2. Definitions and Sources of Metrics Included in Table 1 eAppendix 3. Methods for Multiple Imputation and Multiple Comparisons eFigure 1. Additional Variance Explained by Incorporating Genetic PCs eFigure 2. Correlation of Chronological Age and Epigenetic Aging by Epigenetic Clock: MESA and MBMS eFigure 3. Correlation Matrix for Continuous Variables From MBMS eFigure 4. Correlation Matrix for Continuous Variables From MESA, US-Born eFigure 5. MBMS: Standardized Effect Estimates (Unadjusted) (95% CI) for Early Life Adverse Exposure and Adult Racial Discrimination eFigure 6. MBMS: Standardized Effect Estimates (Unadjusted) (95% CI) for Adult Economic, Pollution, and Segregation Adverse Exposures eFigure 7. MESA: Standardized Effect Estimates (Unadjusted) (95% CI) for Early Life Adverse Exposure and Adult Racial Discrimination eFigure 8. MESA: Standardized Effect Estimates (Unadjusted) (95% CI) for Adult Economic, Pollution, and Segregation Adverse Exposures eFigure 9. Standardized Effect Estimates (Unadjusted) and 95% CIs by Racialized Group for Early Life Adverse Exposures (Reversed Index of Concentration at the Extremes for Racialized Segregatio [file jamanetwopen-e2421832-s001.pdf]

Supplementary Online Content

Krieger N, Testa C, Chen JT, et al. Epigenetic aging and racialized, economic, and environmental injustice: NIMHD social epigenomics program. *JAMA Netw Open*. 2024;7(7):e2421832. doi:10.1001/jamanetworkopen.2024.21832

eTable 1. Primary Citations for and Descriptions of the 10 Epigenetic Clocks Used in This Study

eTable 2. Software Dependency Citations (Using Citations Specified by Each Source)

eTable 3. Number of Overlapping CpG Sites Shared Between Clocks

eTable 4. Chronological Age, Epigenetic Clock and Accelerated Aging Data and Epigenetic Assay Covariates: My Body My Story Study (MBMS; Boston, MA, 2008-2010; Ages 35-64 Years) and Multi-Ethnic Study of Atherosclerosis (MESA; 6 US Sites, Exam 5 Epigenetic Subsample, 2010-2012; Ages 55-94 Years, US-Born)

eTable 5. Standardized Estimates (Unadjusted) of Epigenetic Age Acceleration Associated With Smoking, Age, and Body Mass Index (BMI), for MBMS and MESA US-Born, Estimate and 95% CIs

eAppendix 1. Construction of Surrogate Variables and Genetic Principal Component (PC) Variables

eAppendix 2. Definitions and Sources of Metrics Included in Table 1

eAppendix 3. Methods for Multiple Imputation and Multiple Comparisons

eFigure 1. Additional Variance Explained by Incorporating Genetic PCs

eFigure 2. Correlation of Chronological Age and Epigenetic Aging by Epigenetic Clock: MESA and MBMS

eFigure 3. Correlation Matrix for Continuous Variables From MBMS

eFigure 4. Correlation Matrix for Continuous Variables From MESA, US-Born

eFigure 5. MBMS: Standardized Effect Estimates (Unadjusted) (95% CI) for Early Life Adverse Exposure and Adult Racial Discrimination

eFigure 6. MBMS: Standardized Effect Estimates (Unadjusted) (95% CI) for Adult Economic, Pollution, and Segregation Adverse Exposures

eFigure 7. MESA: Standardized Effect Estimates (Unadjusted) (95% CI) for Early Life Adverse Exposure and Adult Racial Discrimination

eFigure 8. MESA: Standardized Effect Estimates (Unadjusted) (95% CI) for Adult Economic, Pollution, and Segregation Adverse Exposures

eFigure 9. Standardized Effect Estimates (Unadjusted*) and 95% CIs by Racialized Group for Early Life Adverse Exposures (Reversed Index of Concentration at the Extremes for Racialized Segregation at City of Birth) and Adult Individual-, Household-, and Area-Based Adverse Exposures (Employment Status; Housing Tenure, and Reversed Index of Concentration at the Extremes for Income and Housing Tenure): MBMS Participants

eFigure 10. Standardized Effect Estimates (Unadjusted) and 95% CIs by Racialized Group for Early Life Adverse Exposures (Reversed Index of Concentration at the Extremes for Racialized Segregation at City of Birth) and Adult Household- and Area-Based Adverse Exposures (Employment Status; Housing Tenure, and Reversed Index of Concentration at the Extremes for Income and Housing Tenure): MESA Participants

eFigure 11. Standardized Effect Estimates (Unadjusted) by Racialized Group and 95% CIs for Early Life Exposures (Born in a Jim Crow State, Reversed Index of Concentration at the Extremes for Racialized Segregation at City of Birth, State Policy Conservatism in State of Birth, Parents' Education, Participants' Education): MBMS, Not Including Covariate Data on BMI and Smoking

© 2024 Krieger N et al. *JAMA Network Open.*

eFigure 12. Standardized Effect Estimates (Unadjusted) by Racialized Group and 95% CIs for Adult Life Exposures (Set 1: Experiences of Discrimination, Negative log Household Income Ratio to the Poverty Line, Occupational Class, Employment Status, Housing Tenure, and Black Carbon Air Pollution): MBMS, Not Including Covariate Data on BMI and Smoking

eFigure 13. Standardized Effect Estimates (Unadjusted) by Racialized Group and 95% CIs for Adult Life Exposures (Set 2: Pollution Proximity Index [Air Pollution], Reversed Index of Concentration at the Extremes for Income, Racial Segregation, Racialized Economic Segregation, and Housing Tenure): MBMS, Not Including Covariate Data on BMI and Smoking

eFigure 14. Standardized Effect Estimates (Unadjusted) by Racialized Group and 95% CIs for Early Life Exposures (Born in a Jim Crow State, State Policy Conservatism in State of Birth, Parents' Education, Participants' Education): MESA US-Born, Not Including Covariate Data on BMI and Smoking

eFigure 15. Standardized Effect Estimates (Unadjusted) by Racialized Group and 95% CIs for Adult Life Exposures (Set 1: Major Discrimination Scale (racialized), Negative log Household Income Ratio to the Poverty Line, Employment Status, Housing Tenure, and Light Absorption Coefficient [Air Pollution]): MESA US-Born, Not Including Covariate Data on BMI and Smoking

eFigure 16. Standardized Effect Estimates (Unadjusted) by Racialized Group and 95% CIs for Adult Life Exposures (Set 2: Oxides of Nitrogen [Air Pollution], Reversed Index of Concentration at the Extremes for: Income, Racial Segregation, Racialized Economic Segregation, and Housing Tenure): MESA US-Born, Not Including Covariate Data on BMI and Smoking

This supplementary material has been provided by the authors to give readers additional information about their work.

eTable 1. Primary Citations for and Descriptions of the 10 Epigenetic Clocks Used in This Study

Clock	Reference	Generation	n CpGs	n biomarkers	Biomarker details	Training data	Test data	Summary
**Horvath**	Horvath, S. DNA methylation age of human tissues and cell types. Genome Biol. 2013;14:R115.	1st	353	0	NA	Uses 39 datasets, **total n**=3931, **array** = 27k (26) and 450k (13), **sample type** = 27 tissue/cell types	uses 31 datasets, **total n**=3211, **array** = 27k (20) and 450k (11), **sample type** = 22 tissue/cell types	The Horvath clock was designed as a multi-tissue age estimator. 353 CpGs were selected by regressing a transformed version of chronological age on all CpGs, using elastic net. To construct the clock, CpGs are weighted by their regression coefficients, and the average of these is taken to produce an epigenetic age estimate.
**Hannum**	Hannum, G, Guinney, J, Zhao, L, Zhang, L, Hughes, G. Genome-wide methylation profiles reveal quantitative views of human aging rates. Mol Cell. 2013;49:359-367	1st	71	0	NA	**n**=656 (GSE40279) **sample type** = whole blood, **array** = 450k	NA	The Hannum clock is a blood-based age estimator. They used elastic net which selected 71 CpGs (including gender, BMI, diabetes status, ethnicity, and batch in the model). CpGs are weighted by their regression coefficients, and the average of these is taken to produce an epigenetic age estimate.
**EpiToc (Yang)**	Yang, Z, Wong, A, Kuh, D, et al. Correlation of an epigenetic mitotic clock with cancer risk. Genome Biol. 2016;17:205.	1st	385	0	NA	**n**=656 (GSE40279) **sample type** = whole blood, **array** = 450k	GSE42861 (controls only), **n**=335, **sample type** = PBL, **array** = 450k	EpiToc is a mitotic clock indicating the number of stem cell divisions. CpGs are selected by regressing age on DNA methylation sites (adjusting for cell type, batch and sex), and 385 sites which were associated with age at FDR<0.05, within 200bp of transcription start sites, become hypermethylated with age, and marked with PRC2 in HESCs were taken forward to make up the clock. The clock is calculated as the average DNA methylation across the 385 sites.
**Zhang (2019)**	Zhang, Q, Vallerga, CL, Walker, RM, et al. Improved precision of epigenetic clock estimates across tissues and its implication for biological ageing. Genome Med. 2019;11:54.	1st	514	0	NA	**n**=13566 (13 datasets), **sample type**= whole blood (n=13307) + saliva (n=259), **array**=450k and EPIC (reduced to common sites)	**n**=95 (GSE41169), **sample type** = whole blood, **array**=450k	This clock aimed to build a 'perfect' age estimator by using a much larger training dataset. They create two predictors by regressing age on DNA methylation, one using elastic net and one using BLUP; they select elastic net as the most accurate predictor, using 514 CpGs. The clock is built using the weighted average of the CpGs (using model coefficients) plus the model intercept.
**MiAge (Youn and Wang)**	Youn, A, Wang, S. The MiAge Calculator: a DNA methylation-based mitotic age calculator of human tissue types. Epigenetics. 2018;13:192-206.	1st	268	0	NA	**n**=4020 8 TGCA cancer + adjacent normal tissue datasets (BRCA, COAD, HNSC, KIRP, LIHC, PRAD, THCA, UCEC), **array** = 450k, **sample type** = tumour and adjacent tissue	**n**=2221 5 TGCA cancer +adjacent normal tissue (BLCA, KIRC, LUAD, LUSC, and STAD), **array**=450k, **sample type** = tumour and adjacent tissue	MiAge is a mitotic age estimator, estimating cell divisions. 268 DNAm sites that increase with age were selected using a formula that uses probabilities to calculate change from predicted original methylation level at each site (with change estimating the number of cell divisions; increased number of divisions will result in a greater number of somatic replication errors). The clock is calculated by comparing methylation at each CpG to the expected level under the MiAge model, and the resulting clock is the sum of squares of the difference between

							observed and expected methylation levels, summed over all samples at each CpG, and then summed over all CpGs.
**DNAmTL (Lu)**	Lu, AT, Seeboth, A, Tsai, P, et al. DNA methylation-based estimator of telomere length. Aging (Albany NY). 2019;11:5895-5923	140	0	They use telomere length to represent aging	**n**=2256 (718 from WHI and 1538 from JHS), **array** = 450k (WHI) and EPIC (JHS), **sample type** = blood	Test 1: **n**=1078 (100 WHI, 100 JHS, 878 FHS). Test 2: **n** = 9815 (Bogalusa, Twins UK, Lothian Birth cohorts, InCHIANTI), **array** = 450k(FHS, WHI, InCHIANTI, Lothian Birth cohorts, Bogalusa, Twins UK) and EPIC (JHS), **sample type** =blood	DNAmTL is a DNA methylation estimator of telomere length. Leukocyte telomere length was regressed on blood DNA methylation to select 140 CpGs that were associated with telomere length, using elastic net. DNAmTL is constructed by taking the weighted average of these CpGs. The authors find that DNAmTL is more closely related to age than measured telomere length.
**Zhang mortality**	Zhang, Y, Wilson, R, Heiss, 2nd J, et al. DNA methylation signatures in peripheral blood strongly predict all-cause mortality. Nat Commun. 2017;8:14617	514	1	all cause mortality	ESTHER cohort (**n**=954 discovery and 1000 validation). **Sample type** = whole blood, **array** = 450k	KORA cohort (**n**=1727) **sample type** = whole blood, **array** = 450k	The Zhang mortality clock is a mortality predictor, with a higher score indicating a higher risk of mortality. 58 CpGs were found to be associated with all-cause mortality using Cox regression; from these 10 CpGs were selected to comprise the mortality score using LASSO Cox regression. The mortality score is constructed by taking the weighted average of the CpGs (using the LASSO coefficients to weight them).
**PhenoAge (Levine)**	Levine, ME, Lu, AT, Quach, 2nd A, et al. An epigenetic biomarker of aging for lifespan and healthspan. Aging (Albany NY). 2018;10:573-591.	513	10 (selected from 43 using cox penalised regression - hazard of aging related mortality regressed on biomarkers)	Albumin, Creatanine, serum glucose, log CRP, lymphocyte %, MCV (mean red cell volume), red cell distribution width, alkaline phosphatase, white blood cell count, age. **Construction**: parametric proportional hazards model estimating 10 year mortality risk, converted to units of years	<u>phenoage biomarker prediction</u>: NHANES III to select biomarkers (**n**=9926) and NHANES IV to validate (**n**=6209). <u>DNAm site selection</u> in InCHIANTI (**n**=456), sites common to 27k/450k/epic, **sample type** = buffy coat, **array**=450k.	**n** = 7417 (4207 WHI; 2553 FHS; 657 NAS; 1747 JHS). **Array** = 450k (WHS, FHS, NAS), EPIC (JHS). **Sample type** = blood	Phenoage was developed using three steps. Firstly, a composite measure of 'phenotypic age' was constructed by regressing the hazard of aging-related mortality on 42 clinical biomarkers plus chronological age, using Cox penalised regression. Nine biomarkers plus chronological age were selected and in the second step these were combined via a parametric proportional hazards model estimating 10 year mortality risk, converted to units of years to form a phenotypic age estimate. As the final step, this phenotypic age estimate was then regressed on DNA methylation data using elastic net, which selected 513 CGs. The Phenoage estimate of age, in years, is then calculated using the weighted average of the 513 CpGs, using the elastic net coefficients for weighting.

© 2024 Krieger N et al. *JAMA Network Open.*

**DunedinPoAm**	Belsky DW, Caspi A, Arseneault L, Baccarelli A, Corcoran DL, Gao X, et al. Quantification of the pace of biological aging in humans through a blood test, the DunedinPoAm DNA methylation algorithm. eLife. 2020;9.	2nd	46	18 (selected as these were the only ones measured at all 3 timepoints)	HbA1C, Cardiorespiratory Fitness (rev), Waist-hip ratio, FEV1/FVC (rev), FEV1 (rev), Mean arterial pressure, BMI, Leukocyte telomere length (rev), Creatinine clearance (rev), Urea Nitrogen, Lipoprotein(a), Triglycerides, Gum health, Total cholesterol, White blood cell count, hsCRP, HDL cholesterol (rev), ApoB100/ApoA1. **Construction**: all scaled to mean of 0 and SD of 1, with some reversed	**Dunedin cohort. n:** 810. **Sample type:** whole blood. **Array**: 450k	**n**=3019 (1175 US; 771 NAS; 1658 E-Risk; 186 CALERIE) **sample type** = whole blood, **array** = 450k (NAS, E-Risk), EPIC (US, CALERIE)	DunedinPoAm was created by firstly modelling rate of change in 18 biomarkers over 12 years in the same individuals. The rates of change were combined into a composite measure termed 'Pace-of-Aging'. Then elastic net was used to select CpG sites that predict this 'Pace-of-Aging' measure.
**GrimAge**	Lu, AT, Quach, A, Wilson, JG, et al. DNA methylation GrimAge strongly predicts lifespan and healthspan. Aging (Albany NY). 2019;11:303-327.	2nd	1030	7 (selected from 88 as they are physiological risk factors and stress factors, and had DNAm sites in elastic net, and are predictive of mortality), plus age sex and DNAm smoking pack-years	chronological age (Age), sex (Female), and DNAm based surrogates for smoking pack-years (DNAm PACKYRS), adrenomedullin levels (DNAm ADM), beta-2 microglobulin (DNAm B2M), cystatin C (DNAm Cystatin C), growth differentiation factor 15 (DNAm GDF-15), leptin (DNAm Leptin), plasminogen activation inhibitor 1 (DNAm PAI-1), tissue inhibitor metalloproteinase 1 (DNAm TIMP-1).	FHS offspring study: training subset **n**=1731. **Sample type** = buffy coat, **array** = 450k	**n**=7375 (FHS offspring study test subset n=625, WHI BA23 (N=2107), WHI EMPC (N=1972), JHS (N=1747), and InChianti study (N=924)), **sample type** = blood, **array**= 450k (FHS, WHI, InCHIANTI) and EPIC (JHS)	GrimAge was created using a two step process. In the first step, elastic net was used to identify DNAm sites that could be used as surrogate markers for 88 plasma proteins and smoking pack years (including chronological age and sex in the models). 12 of these surrogate markers plus smoking pack years were sufficiently accurate in training and test datasets. The second step regresses time to death (all cause mortality) on chronological age, sex, 12 DNAm surrogate protein markers and DNAm markers for smoking pack years; 7 DNAm markers plus age, sex and the pack-years DNAm markers were selected by the model, then the linear combination of the selected variables is transformed into an age estimate.

Note: for reproducibility, we have made an R script containing calculations for all clocks except GrimAge available on GitHub, at: https://github.com/shwatkins/epigenetic_clocks_mbms_mesa .

© 2024 Krieger N et al. *JAMA Network Open.*

eTable 2. Software Dependency Citations (Using Citations Specified by Each Source)

1.	R Core Team (2022). R: A language and environment for statistical computing. R Foundation for Statistical Computing, Vienna, Austria. URL https://www.R-project.org/.
2.	Ou J (2021). _colorBlindness: Safe Color Set for Color Blindness. R package version 0.1.9, <https://CRAN.R-project.org/package=colorBlindness>.
3.	Firke S (2021). _janitor: Simple Tools for Examining and Cleaning Dirty Data_. R package version 2.1.0, <https://CRAN.R-project.org/package=janitor>.
4.	Bache S, Wickham H (2022). _magrittr: A Forward-Pipe Operator for R_. R package version 2.0.3, <https://CRAN.R-project.org/package=magrittr>.
5.	Hadley Wickham. testthat: Get Started with Testing. *The R Journal*, vol. 3, no. 1, pp. 5--10, 2011
6.	Wickham H, Averick M, Bryan J, Chang W, McGowan LD, François R, Grolemund G, Hayes A, Henry L, Hester J, Kuhn M, Pedersen TL, Miller E, Bache SM, Müller K, Ooms J, Robinson D, Seidel DP, Spinu V, Takahashi K, Vaughan D, Wilke C, Woo K, Yutani H (2019). "Welcome to the tidyverse." *Journal of Open Source Software*, *4*(43), 1686. doi:10.21105/joss.01686 <https://doi.org/10.21105/joss.01686>.
7.	Robinson D, Hayes A, Couch S (2022). _broom: Convert Statistical Objects into Tidy Tibbles. R package version 1.0.2, <https://CRAN.R-project.org/package=broom>.
8.	Bolker B, Robinson D (2022). _broom.mixed: Tidying Methods for Mixed Models. R package version 0.2.9.4, <https://CRAN.R-project.org/package=broom.mixed>.
9.	Stef van Buuren, Karin Groothuis-Oudshoorn (2011). mice: Multivariate Imputation by Chained Equations in R. *Journal of Statistical Software*, 45(3), 1-67. DOI 10.18637/jss.v045.i03.
10.	Douglas Bates, Martin Maechler, Ben Bolker, Steve Walker (2015). Fitting Linear Mixed-Effects Models Using lme4. *Journal of Statistical Software,* 67(1), 1-48. doi:10.18637/jss.v067.i01.
11.	Müller K (2020). _here: A Simpler Way to Find Your Files. R package version 1.0.1, <https://CRAN.R-project.org/package=here>.
12.	Schauberger P, Walker A (2022). _openxlsx: Read, Write and Edit xlsx Files. R package version 4.2.5.1, <https://CRAN.R-project.org/package=openxlsx>.
13.	Hadley Wickham, Jennifer Bryan. R Packages (2e). O'Reilly; Available from: https://r-pkgs.org/
14.	JJ Allaire and Yihui Xie and Jonathan McPherson and Javier Luraschi and Kevin Ushey and Aron Atkins and Hadley Wickham and Joe Cheng and Winston Chang and Richard Iannone (2022). rmarkdown: Dynamic Documents for R. R package version 2.19. URL https://rmarkdown.rstudio.com.
15.	Leek JT, Johnson WE, Parker HS, Fertig EJ, Jaffe AE, Zhang Y, Storey JD, Torres LC (2023). *sva: Surrogate Variable Analysis*. R package version 3.48.0.

© 2024 Krieger N et al. *JAMA Network Open.*

eTable 3. Number of Overlapping CpG Sites Shared Between Clocks

	Horvath (1st)	Hannum (1st)	epiToc (1st)	Zhang Age (1st)	MiAge (1st)	DNAmTL (2nd)	Zhang Mortality (2nd)	PhenoAge (2nd)	Dunedin (2nd)
Horvath (1st)	353	6	1	11	0	0	0	41	0
Hannum (1st)	6	71	0	30	0	5	0	6	0
epiToc (1st)	1	0	385	0	2	0	0	1	0
Zhang Age (1st)	11	30	0	515	1	6	0	16	0
MiAge (1st)	0	0	2	1	268	0	0	0	0
DNAmTL (2nd)	0	5	0	6	0	140	0	1	0
Zhang Mortality (2nd)	0	0	0	0	0	0	10	0	1
PhenoAge (2nd)	41	6	1	16	0	1	0	514	0
Dunedin (2nd)	0	0	0	0	0	0	1	0	46

© 2024 Krieger N et al. *JAMA Network Open.*

**eTable 4. Chronological Age, Epigenetic Clock and Accelerated Aging Data and Epigenetic Assay Covariates: My Body My Story Study (MBMS; Boston, MA, 2008-2010; Ages 35-64 Years) and Multi-Ethnic Study of Atherosclerosis (MESA; 6 US Sites, Exam 5 Epigenetic Subsample, 2010-2012; Ages 55-94 Years, US-Born)**					
**Variable**	**Chronological age, epigenetic clocks and measures of accelerated aging[a]: mean (SD [standard deviation])**				
	**My Body My Story (MBMS) (N = 293)[b]**		**Multi-Ethnic Study of Atherosclerosis (MESA, Exam 5) (N = 975)[b]**		
	**Black non-Hispanic**	**White non-Hispanic**	**Black non-Hispanic**	**White non-Hispanic**	**Hispanic**
**Total: N**	224	69	229	555	191
**Chronological age: mean (SD)**	49.0 (7.8)	48.7 (8.3)	71.0 (8.9)	70.1 (9.5)	68.4 (8.9)
**Raw estimates: mean (SD)**					
**1st Generation**					
**-- Age estimator**					
Hannum[c]	41.66 (6.78)	43.13 (7.44)	72.26 (8.00)	74.27 (8.62)	72.39 (8.53)
Horvath[c]	50.23 (7.49)	49.41 (8.82)	64.58 (8.14)	65.13 (8.43)	62.03 (9.34)
Zhang (age)	48.62 (7.52)	49.60 (8.83)	70.89 (7.31)	71.92 (7.92)	69.58 (7.47)
**-- Mitotic age**					
MiAge[c]	820.59 (143.34)	766.72 (104.52)	770.84 (101.45)	718.46 (81.05)	736.97 (100.17)
EpiToc[c]	0.06 (0.02)	0.06 (0.01)	0.09 (0.01)	0.09 (0.00)	0.09 (0.01)
**-- Telomere length**					
DNAmTL	-0.84 (0.25)	-0.96 (0.24)	-1.02 (0.19)	-1.19 (0.21)	-1.14 (0.23)
**2nd Generation**					
**-- Age estimator**					
Phenoage	42.13 (7.93)	40.80 (8.62)	72.52 (9.12)	72.30 (9.21)	71.35 (9.48)
DunedinPoAm (pace of aging)	1.14 (0.09)	1.10 (0.10)	1.10 (0.08)	1.05 (0.07)	1.06 (0.07)
**-- Mortality predictor**					
Zhang (mortality) [c]	-1.15 (0.44)	-1.18 (0.43)	-1.88 (0.38)	-1.84 (0.33)	-1.92 (0.36)
GrimAge	54.77 (6.96)	53.56 (7.82)	81.52 (7.64)	79.52 (8.04)	78.73 (8.29)
**Accelerated aging estimators (detrended for age): mean (SD)**					
**1st Generation**					
**-- Age estimator**					
Hannum[c]	-0.39 (4.59)	1.26 (5.84)	-1.74 (4.97)	0.94 (4.44)	0.34 (4.91)
Horvath[c]	0.15 (5.85)	-0.50 (7.27)	-0.25 (5.20)	0.93 (5.00)	-0.96 (6.53)
Zhang (age)	-0.28 (4.27)	0.92 (6.12)	-0.89 (3.08)	0.82 (3.01)	-0.24 (2.99)
**-- Mitotic age**					
MiAge[c]	12.48 (140.50)	-40.52 (105.25)	33.35 (100.97)	-17.76 (80.36)	3.17 (97.43)
EpiToc[c]	0.00 (0.02)	0.00 (0.01)	0.00 (0.01)	0.00 (0.00)	0.00 (0.01)
**-- Telomere length**					
DNAmTL	0.03 (0.23)	-0.09 (0.21)	0.12 (0.16)	-0.06 (0.17)	-0.03 (0.20)
**2nd Generation**					
**-- Age estimator**					
Phenoage	0.28 (6.37)	-0.89 (7.57)	-0.31 (6.29)	0.13 (5.86)	0.45 (5.83)
DunedinPoAm	N/A	N/A	N/A	N/A	N/A
**-- Mortality predictor**					
Zhang (mortality) [c]	0.01 (0.44)	-0.03 (0.43)	0.00 (0.38)	0.05 (0.33)	-0.01 (0.35)
GrimAge	0.24 (4.71)	-0.79 (5.07)	1.10 (4.55)	-0.24 (4.10)	0.20 (4.04)
**Epigenetic accelerated aging[d]**					
Epigenetic accelerated aging on one or more age estimator clocks: N (%)	204 (91.1%)	61 (88.4%)	227 (99.1%)	554 (99.8%)	191 (100%)
Epigenetic accelerated aging on all 5 age estimator clocks: N (%)	7 (3.1%)	9 (13%)	23 (10%)	17 (8.9%)	84 (15.1%)
Epigenetic accelerated aging on Hannum: N (%)	13 (5.8%)	13 (18.8%)	135 (59%)	456 (82.2%)	153 (80.1%)
Epigenetic accelerated aging on Horvath: N (%)	130 (58%)	40 (58%)	31 (13.5%)	94 (16.9%)	21 (11%)
Epigenetic accelerated aging on Phenoage: N (%)	33 (14.7%)	17 (24.6%)	141 (61.6%)	358 (64.5%)	136 (71.2%)
Epigenetic accelerated aging on Zhang: N (%)	98 (43.8%)	42 (60.9%)	106 (46.3%)	382 (68.8%)	122 (63.9%)
Epigenetic accelerated aging on DNAGrimAge: N (%)	188 (83.9%)	52 (75.4%)	27 (99.1%)	552 (99.5%)	191 (100%)
**Predicted cell type proportion estimates: mean (SD)[e]**					
B cell	0.08 (0.02)	0.06 (0.01)	0.04 (0.03)	0.03 (0.02)	0.03 (0.02)
CD4T	0.17 (0.05)	0.15 (0.04)	0.04 (0.02)	0.03 (0.03)	0.03 (0.01)

© 2024 Krieger N et al. *JAMA Network Open.*

CD8T	0.01 (0.02)	0.00 (0.01)	0.00 (0.01)	0.00 (0.00)	0.00 (0.00)
Eosinophil	0.01 (0.02)	0.00 (0.01)	0.01 (0.01)	0.01 (0.01)	0.01 (0.01)
Monocyte	0.12 (0.02)	0.12 (0.02)	0.90 (0.05)	0.91 (0.04)	0.92 (0.04)
Neutrophil	0.55 (0.10)	0.62 (0.08)	0.00 (0.00)	0.00 (0.01)	0.00 (0.01)
Natural Killer	0.11 (0.04)	0.09 (0.04)	0.01 (0.01)	0.01 (0.01)	0.01 (0.01)

[a] The raw data are the values of the epigenetic clocks, not detrended for age. Accelerated aging is detrended for age and refers to the residuals when epigenetic age is regressed on chronological age for each of the MBMS and MESA populations; an accelerated aging value greater than 0 indicates the participant's epigenetic clock value exceeded the predicted epigenetic clock value for that participant given their chronological age. All models controlled for the specified covariates (age, sex/gender, cell-type proportions, smoking, BMI, and surrogate variables).

[b] MBMS inclusion criteria were that all participants had to self-identify as being US-born and being either Black non-Hispanic or white non-Hispanic; MESA did not have these nativity restrictions, and in the Exam 5 epigenetic subsample, 77.1% were US-born and 22.7% were born outside of the US, and we report solely on the US-born MESA participants..

[c] These clocks contain CpG sites which are not available on the EPIC array, consequently their values in MBMS are based on the available subset of clock CpG sites.

[d] We present only the data for the epigenetic clock measures that are interpretable on the age (years) scale: Horvath, Hannum, Zhang, PhenoAge, GrimAge); among the 1268 participants (MBMS + MESA), 11.0% exhibited epigenetic accelerated for all 5 of these epigenetic clocks. For these epigenetic clocks, age acceleration (based on detrending the raw clock measures for chronological age) is interpretable as years of age acceleration (relative to chronological age), and the SDs are on the same scale (years). We do not include the data for the epigenetic clocks that are not on the years of age scale (EpiToc, MiAge, DNAmTL, Zhang mortality, and DunedinPoAm), since interpretation is specific to the clock (e.g. EpiToc and MiAge are on the scale of cell divisions, Zhang is based on a predicted risk score for mortality, and DunedinPoAm is based on rate of change in biomarkers longitudinally).

[e] Cell type proportions for blood samples were estimated from DNA methylation levels measured in the samples and in purified cell types {Houseman, E. A. et al. DNA methylation arrays as surrogate measures of cell mixture distribution. *BMC Bioinformatics* 13, 86 (2012); Reinius, L. E. et al. Differential DNA methylation in purified human blood cells: implications for cell lineage and studies on disease susceptibility. *PLoS ONE* 7, e41361 (2012)] In MESA, samples were purified using flow cytometry to contain >90% monocytes [Reynolds LM, Taylor JR, Ding J, et al. Age-related variations in the methylome associated with gene expression in human monocytes and T cells. *Nat Commun*. 2014;5:5366. Published 2014 Nov 18. doi:10.1038/ncomms6366], consistent with our estimates showing 91% monocyte content on average.

© 2024 Krieger N et al. *JAMA Network Open.*

eTable 5. Standardized Estimates (Unadjusted) of Epigenetic Age Acceleration Associated With Smoking, Age, and Body Mass Index (BMI), for MBMS and MESA US-Born, Estimate and 95% CIs

A. Standardized Estimates (Unadjusted*) of Epigenetic Age Acceleration Associated With Smoking (Current in Last 8 Hours vs Never), MBMS, Estimate and 95% CIs

Clock	Born in a Jim Crow State	Reversed ICE for Racialized Segregation at City of Birth (Black vs. White)	State Policy Conservatism in State of Birth	Parent's Highest Education	Participant's Highest Education	Experiences of Discrimination	Negative log10(household income / poverty line) in 2010 Dollars	Occupational Class	Housing Tenure — Paying Mortgage	Black Carbon	Pollution Proximity Index	Reversed Residential Census Tract ICE for Income	Reversed Residential Census Tract ICE for Racialized Economic Segregation	Reversed Residential Census Tract ICE for Racial Segregation	Reversed Residential Census Tract ICE for Housing Tenure
Horvath (1st)	0.2 (0.0, 0.4)	0.2 (0.0, 0.4)	0.2 (0.0, 0.4)	0.2 (-0.1, 0.4)	0.2 (0.0, 0.4)	0.2 (0.0, 0.4)	0.2 (0.0, 0.4)	0.2 (0.0, 0.4)	0.2 (0.0, 0.4)	0.2 (0.0, 0.4)	0.2 (0.0, 0.4)	0.2 (0.0, 0.4)	0.2 (0.0, 0.4)	0.2 (0.0, 0.4)	0.2 (0.0, 0.4)
Hannum (1st)	0.2 (0.0, 0.4)	0.2 (0.0, 0.4)	0.2 (0.0, 0.4)	0.3 (0.0, 0.5)	0.2 (0.0, 0.4)	0.2 (0.0, 0.3)	0.2 (0.0, 0.3)	0.2 (0.0, 0.4)	0.2 (0.0, 0.4)	0.2 (0.0, 0.4)	0.2 (0.0, 0.4)	0.2 (0.0, 0.4)	0.2 (0.0, 0.4)	0.2 (0.0, 0.3)	0.2 (0.0, 0.4)
epiToc (1st)	0.3 (0.1, 0.4)	0.3 (0.1, 0.4)	0.3 (0.1, 0.4)	0.4 (0.2, 0.6)	0.3 (0.1, 0.4)	0.3 (0.1, 0.4)	0.3 (0.1, 0.4)	0.3 (0.1, 0.4)	0.3 (0.1, 0.5)	0.3 (0.1, 0.4)	0.3 (0.1, 0.4)	0.3 (0.1, 0.4)	0.3 (0.1, 0.4)	0.3 (0.1, 0.4)	0.3 (0.1, 0.5)
Zhang Age (1st)	0.1 (-0.1, 0.3)	0.1 (-0.1, 0.3)	0.1 (-0.1, 0.3)	0.1 (-0.1, 0.3)	0.1 (-0.1, 0.3)	0.1 (-0.1, 0.3)	0.1 (-0.1, 0.3)	0.1 (-0.1, 0.3)	0.1 (-0.1, 0.3)	0.1 (-0.1, 0.3)	0.1 (-0.1, 0.3)	0.1 (-0.1, 0.3)	0.1 (-0.1, 0.3)	0.1 (-0.1, 0.3)	0.1 (-0.1, 0.3)
MiAge (1st)	0.2 (0.1, 0.4)	0.2 (0.1, 0.4)	0.2 (0.1, 0.4)	0.3 (0.1, 0.5)	0.2 (0.1, 0.4)	0.2 (0.1, 0.4)	0.2 (0.1, 0.4)	0.2 (0.1, 0.4)	0.3 (0.1, 0.4)	0.2 (0.1, 0.4)	0.2 (0.1, 0.4)	0.2 (0.1, 0.4)	0.2 (0.1, 0.4)	0.2 (0.1, 0.4)	0.3 (0.1, 0.4)
DNAmTL (1st)	0.3 (0.1, 0.5)	0.3 (0.1, 0.5)	0.3 (0.1, 0.5)	0.3 (0.0, 0.5)	0.2 (0.0, 0.4)	0.3 (0.1, 0.5)	0.3 (0.1, 0.5)	0.3 (0.0, 0.5)	0.3 (0.1, 0.5)	0.3 (0.1, 0.5)	0.3 (0.1, 0.5)	0.3 (0.1, 0.5)	0.3 (0.0, 0.5)	0.2 (0.0, 0.5)	0.3 (0.1, 0.5)
Zhang Mortality (2nd)	0.6 (0.4, 0.8)	0.6 (0.4, 0.8)	0.6 (0.4, 0.8)	0.6 (0.4, 0.8)	0.5 (0.4, 0.7)	0.6 (0.4, 0.8)	0.6 (0.4, 0.8)	0.6 (0.4, 0.8)	0.6 (0.4, 0.7)	0.6 (0.4, 0.8)	0.6 (0.4, 0.8)	0.5 (0.3, 0.7)	0.5 (0.3, 0.7)	0.6 (0.4, 0.7)	0.6 (0.4, 0.8)
PhenoAge (2nd)	0.3 (0.1, 0.5)	0.3 (0.1, 0.5)	0.3 (0.1, 0.5)	0.3 (0.0, 0.5)	0.3 (0.1, 0.5)	0.3 (0.1, 0.5)	0.3 (0.1, 0.5)	0.3 (0.1, 0.5)	0.3 (0.1, 0.5)	0.3 (0.1, 0.5)	0.3 (0.1, 0.5)	0.3 (0.1, 0.5)	0.3 (0.1, 0.5)	0.3 (0.1, 0.5)	0.3 (0.1, 0.5)
DunedinPoAm (2nd)	0.7 (0.5, 0.9)	0.7 (0.5, 0.9)	0.7 (0.5, 0.9)	0.7 (0.4, 0.9)	0.7 (0.5, 0.9)	0.7 (0.5, 0.9)	0.7 (0.5, 0.9)	0.7 (0.5, 0.9)	0.7 (0.5, 0.9)	0.7 (0.5, 0.9)	0.7 (0.5, 0.9)	0.7 (0.4, 0.9)	0.7 (0.5, 0.9)	0.7 (0.5, 0.9)	0.7 (0.5, 0.9)
GrimAge (2nd)	0.6 (0.5, 0.8)	0.6 (0.5, 0.8)	0.6 (0.5, 0.8)	0.6 (0.4, 0.8)	0.6 (0.5, 0.8)	0.6 (0.5, 0.8)	0.6 (0.5, 0.8)	0.6 (0.5, 0.8)	0.6 (0.5, 0.8)	0.6 (0.5, 0.8)	0.6 (0.5, 0.8)	0.6 (0.5, 0.8)	0.6 (0.5, 0.8)	0.6 (0.5, 0.8)	0.6 (0.5, 0.8)

B. Standardized Estimates (Unadjusted*) of Epigenetic Age Acceleration Associated With Smoking (Current vs Never), MESA US-Born, Estimate and 95% CIs

Clock	Born in a Jim Crow State	State Policy Conservatism in State of Birth	Parent's Highest Education	Participant's Highest Education	Major Discrimination Scale	Negative log10(household income / poverty line) in 2010 Dollars	Occupational Class	Housing Tenure — Paying Mortgage	Light Absorption Coefficient	Nitrous Oxides	Reversed Residential Census Tract ICE for Income	Reversed Residential Census Tract ICE for Racialized Economic Segregation	Reversed Residential Census Tract ICE for Racial Segregation	Reversed Residential Census Tract ICE for Housing Tenure
Horvath (1st)	0.0 (-0.1, 0.2)	0.0 (-0.1, 0.2)	0.1 (-0.1, 0.2)	0.0 (-0.1, 0.2)	0.0 (-0.1, 0.2)	0.1 (-0.1, 0.2)	0.1 (-0.1, 0.2)	0.0 (-0.1, 0.2)	0.0 (-0.1, 0.2)	0.0 (-0.1, 0.2)	0.1 (-0.1, 0.2)	0.1 (-0.1, 0.2)	0.1 (-0.1, 0.2)	0.1 (0.0, 0.2)
Hannum (1st)	0.0 (-0.1, 0.1)	0.0 (-0.1, 0.1)	0.0 (-0.1, 0.1)	0.0 (-0.1, 0.1)	0.0 (-0.1, 0.1)	0.0 (-0.1, 0.1)	0.0 (-0.1, 0.1)	0.0 (-0.1, 0.1)	0.0 (-0.1, 0.1)	0.0 (-0.1, 0.1)	0.0 (-0.1, 0.1)	0.0 (-0.1, 0.1)	0.0 (-0.1, 0.1)	0.0 (-0.1, 0.1)
epiToc (1st)	0.0 (-0.2, 0.2)	0.0 (-0.1, 0.2)	0.0 (-0.1, 0.2)	0.0 (-0.2, 0.1)	0.0 (-0.2, 0.2)	0.0 (-0.2, 0.1)	0.0 (-0.1, 0.2)	0.0 (-0.2, 0.1)	0.0 (-0.2, 0.2)	0.0 (-0.2, 0.1)	0.0 (-0.2, 0.2)	0.0 (-0.2, 0.1)	0.0 (-0.2, 0.1)	0.0 (-0.1, 0.2)
Zhang Age (1st)	-0.1 (-0.1, 0.0)	-0.1 (-0.1, 0.0)	-0.1 (-0.1, 0.0)	-0.1 (-0.1, 0.0)	-0.1 (-0.1, 0.0)	-0.1 (-0.1, 0.0)	-0.1 (-0.2, 0.0)	-0.1 (-0.2, 0.0)	-0.1 (-0.1, 0.0)	-0.1 (-0.1, 0.0)	-0.1 (-0.1, 0.0)	-0.1 (-0.1, 0.0)	-0.1 (-0.1, 0.0)	-0.1 (-0.1, 0.0)
MiAge (1st)	-0.1 (-0.3, 0.0)	-0.1 (-0.3, 0.0)	-0.1 (-0.3, 0.0)	-0.1 (-0.3, 0.0)	-0.1 (-0.3, 0.0)	-0.1 (-0.3, 0.0)	-0.1 (-0.3, 0.0)	-0.1 (-0.3, 0.0)	-0.1 (-0.3, 0.0)	-0.1 (-0.3, 0.0)	-0.1 (-0.3, 0.0)	-0.1 (-0.3, 0.0)	-0.1 (-0.3, 0.0)	-0.1 (-0.3, 0.0)
DNAmTL (1st)	0.3 (0.1, 0.4)	0.3 (0.1, 0.4)	0.2 (0.1, 0.4)	0.2 (0.1, 0.4)	0.3 (0.1, 0.4)	0.2 (0.1, 0.4)	0.2 (0.1, 0.4)	0.2 (0.1, 0.4)	0.3 (0.1, 0.4)	0.3 (0.1, 0.4)	0.2 (0.1, 0.4)	0.2 (0.1, 0.4)	0.3 (0.1, 0.4)	0.3 (0.1, 0.4)
Zhang Mortality (2nd)	1.6 (1.4, 1.8)	1.6 (1.4, 1.7)	1.6 (1.4, 1.8)	1.6 (1.4, 1.7)	1.6 (1.4, 1.7)	1.6 (1.4, 1.7)	1.6 (1.4, 1.7)	1.6 (1.4, 1.7)	1.6 (1.4, 1.7)	1.6 (1.4, 1.7)	1.6 (1.4, 1.7)	1.6 (1.4, 1.8)	1.6 (1.4, 1.8)	1.6 (1.4, 1.8)
PhenoAge (2nd)	0.1 (0.0, 0.3)	0.1 (0.0, 0.3)	0.1 (0.0, 0.3)	0.1 (0.0, 0.3)	0.1 (0.0, 0.3)	0.1 (0.0, 0.3)	0.1 (0.0, 0.3)	0.1 (0.0, 0.3)	0.1 (0.0, 0.3)	0.1 (0.0, 0.3)	0.1 (0.0, 0.3)	0.1 (0.0, 0.3)	0.1 (0.0, 0.3)	0.1 (0.0, 0.3)
DunedinPoAm (2nd)	1.7 (1.5, 1.9)	1.7 (1.5, 1.9)	1.7 (1.5, 1.9)	1.7 (1.5, 1.8)	1.7 (1.5, 1.9)	1.7 (1.5, 1.8)	1.7 (1.5, 1.8)	1.7 (1.5, 1.9)	1.7 (1.5, 1.9)	1.7 (1.5, 1.9)	1.7 (1.5, 1.8)	1.7 (1.5, 1.8)	1.7 (1.5, 1.9)	1.7 (1.5, 1.8)
GrimAge (2nd)	1.0 (0.9, 1.0)	1.0 (0.9, 1.0)	1.0 (0.9, 1.0)	1.0 (0.9, 1.0)	1.0 (0.9, 1.0)	0.9 (0.9, 1.0)	1.0 (0.9, 1.0)	1.0 (0.9, 1.0)	1.0 (0.9, 1.0)	1.0 (0.9, 1.1)	1.0 (0.9, 1.0)	1.0 (0.9, 1.0)	1.0 (0.9, 1.0)	1.0 (0.9, 1.0)

© 2024 Krieger N et al. *JAMA Network Open.*

C. Standardized Estimates (Unadjusted*) of Epigenetic Age Acceleration Associated With Age, MBMS, Estimate and 95% CIs

Clock	Born in a Jim Crow State	Reversed ICE for Racialized Segregation at City of Birth (Black vs. White)	State Policy Conservatism in State of Birth	Parent's Highest Education	Participant's Highest Education	Experiences of Discrimination	Negative log10(household income / poverty line) in 2010 Dollars	Occupational Class	Housing Tenure — Paying Mortgage	Black Carbon	Pollution Proximity Index	Reversed Residential Census Tract ICE for Income	Reversed Residential Census Tract ICE for Racialized Economic Segregation	Reversed Residential Census Tract ICE for Racial Segregation	Reversed Residential Census Tract ICE for Housing Tenure
Horvath (1st)	0.6 (0.5, 0.7)	0.6 (0.5, 0.7)	0.6 (0.5, 0.7)	0.6 (0.5, 0.7)	0.6 (0.5, 0.7)	0.6 (0.5, 0.7)	0.6 (0.5, 0.7)	0.6 (0.5, 0.7)	0.6 (0.5, 0.7)	0.6 (0.5, 0.7)	0.6 (0.5, 0.7)	0.6 (0.5, 0.7)	0.6 (0.5, 0.7)	0.6 (0.5, 0.7)	0.6 (0.5, 0.7)
Hannum (1st)	0.6 (0.5, 0.7)	0.6 (0.6, 0.7)	0.6 (0.5, 0.7)	0.7 (0.6, 0.8)	0.6 (0.6, 0.7)	0.6 (0.6, 0.7)	0.6 (0.6, 0.7)	0.6 (0.6, 0.7)	0.6 (0.6, 0.7)	0.6 (0.6, 0.7)	0.7 (0.6, 0.7)	0.7 (0.6, 0.7)	0.6 (0.6, 0.7)	0.6 (0.6, 0.7)	0.7 (0.6, 0.7)
epiToc (1st)	0.1 (0.0, 0.2)	0.1 (0.1, 0.2)	0.1 (0.0, 0.2)	0.1 (0.1, 0.2)	0.1 (0.1, 0.2)	0.1 (0.1, 0.2)	0.1 (0.1, 0.2)	0.1 (0.1, 0.2)	0.1 (0.1, 0.2)	0.1 (0.1, 0.2)	0.1 (0.1, 0.2)	0.1 (0.1, 0.2)	0.1 (0.1, 0.2)	0.1 (0.1, 0.2)	0.1 (0.1, 0.2)
Zhang Age (1st)	0.7 (0.7, 0.8)	0.8 (0.7, 0.8)	0.7 (0.7, 0.8)	0.8 (0.7, 0.8)	0.8 (0.7, 0.8)	0.8 (0.7, 0.8)	0.8 (0.7, 0.8)	0.8 (0.7, 0.8)	0.8 (0.7, 0.8)	0.8 (0.7, 0.8)	0.8 (0.7, 0.8)	0.8 (0.7, 0.8)	0.8 (0.7, 0.8)	0.8 (0.7, 0.8)	0.8 (0.7, 0.8)
MiAge (1st)	0.1 (0.0, 0.2)	0.1 (0.1, 0.2)	0.1 (0.0, 0.2)	0.1 (0.0, 0.2)	0.1 (0.1, 0.2)	0.1 (0.1, 0.2)	0.1 (0.1, 0.2)	0.1 (0.1, 0.2)	0.1 (0.1, 0.2)	0.1 (0.1, 0.2)	0.1 (0.1, 0.2)	0.1 (0.1, 0.2)	0.1 (0.1, 0.2)	0.1 (0.1, 0.2)	0.1 (0.1, 0.2)
DNAmTL (1st)	0.3 (0.2, 0.4)	0.4 (0.3, 0.5)	0.3 (0.3, 0.4)	0.4 (0.3, 0.5)	0.4 (0.3, 0.4)	0.4 (0.3, 0.5)	0.4 (0.3, 0.5)	0.4 (0.3, 0.4)	0.4 (0.3, 0.4)	0.4 (0.3, 0.5)	0.4 (0.3, 0.4)	0.4 (0.3, 0.4)	0.4 (0.3, 0.4)	0.4 (0.3, 0.4)	0.4 (0.3, 0.5)
Zhang Mortality (2nd)	0.0 (-0.1, 0.1)	0.0 (-0.1, 0.1)	0.0 (-0.1, 0.1)	0.1 (0.0, 0.1)	0.0 (-0.1, 0.1)	0.0 (-0.1, 0.1)	0.0 (0.0, 0.1)	0.0 (0.0, 0.1)	0.0 (-0.1, 0.1)	0.0 (-0.1, 0.1)	0.0 (-0.1, 0.1)	0.0 (-0.1, 0.1)	0.0 (-0.1, 0.1)	0.0 (-0.1, 0.1)	0.0 (-0.1, 0.1)
PhenoAge (2nd)	0.6 (0.5, 0.6)	0.6 (0.5, 0.7)	0.6 (0.5, 0.7)	0.6 (0.5, 0.7)	0.6 (0.5, 0.7)	0.6 (0.5, 0.7)	0.6 (0.5, 0.7)	0.6 (0.5, 0.7)	0.6 (0.5, 0.7)	0.6 (0.5, 0.7)	0.6 (0.5, 0.7)	0.6 (0.5, 0.7)	0.6 (0.5, 0.7)	0.6 (0.5, 0.7)	0.6 (0.5, 0.7)
DunedinPoAm (2nd)	-0.1 (-0.2, 0.0)	0.0 (-0.1, 0.1)	-0.1 (-0.2, 0.0)	0.0 (-0.1, 0.1)	0.0 (-0.1, 0.1)	0.0 (-0.1, 0.1)	0.0 (-0.1, 0.1)	0.0 (-0.1, 0.1)	0.0 (-0.1, 0.1)	0.0 (-0.1, 0.1)	0.0 (-0.1, 0.1)	0.0 (-0.1, 0.1)	0.0 (-0.1, 0.1)	0.0 (-0.1, 0.1)	0.0 (-0.1, 0.1)
GrimAge (2nd)	0.7 (0.7, 0.8)	0.7 (0.7, 0.8)	0.7 (0.7, 0.8)	0.7 (0.7, 0.8)	0.7 (0.7, 0.8)	0.7 (0.7, 0.8)	0.7 (0.7, 0.8)	0.7 (0.7, 0.8)	0.7 (0.7, 0.8)	0.7 (0.7, 0.8)	0.7 (0.7, 0.8)	0.7 (0.7, 0.8)	0.7 (0.7, 0.8)	0.7 (0.7, 0.8)	0.7 (0.7, 0.8)

D. Standardized Estimates (Unadjusted*) of Epigenetic Age Acceleration Associated With Age, MESA US-Born, Estimate and 95% CIs

Clock	Born in a Jim Crow State	State Policy Conservatism in State of Birth	Parent's Highest Education	Participant's Highest Education	Major Discrimination Scale	Negative log10(household income / poverty line) in 2010 Dollars	Occupational Class	Housing Tenure — Paying Mortgage	Light Absorption Coefficient	Nitrous Oxides	Reversed Residential Census Tract ICE for Income	Reversed Residential Census Tract ICE for Racialized Economic Segregation	Reversed Residential Census Tract ICE for Racial Segregation	Reversed Residential Census Tract ICE for Housing Tenure
Horvath (1st)	0.5 (0.5, 0.6)	0.6 (0.5, 0.6)	0.6 (0.5, 0.6)	0.5 (0.5, 0.6)	0.5 (0.5, 0.6)	0.6 (0.5, 0.6)	0.5 (0.5, 0.6)	0.6 (0.5, 0.6)	0.5 (0.5, 0.6)	0.5 (0.5, 0.6)	0.5 (0.5, 0.6)	0.5 (0.5, 0.6)	0.5 (0.5, 0.6)	0.5 (0.5, 0.6)
Hannum (1st)	0.6 (0.6, 0.7)	0.6 (0.6, 0.7)	0.6 (0.6, 0.7)	0.6 (0.6, 0.7)	0.6 (0.6, 0.7)	0.7 (0.6, 0.7)	0.6 (0.6, 0.7)	0.7 (0.6, 0.7)	0.6 (0.6, 0.7)	0.6 (0.6, 0.7)	0.6 (0.6, 0.7)	0.6 (0.6, 0.7)	0.6 (0.6, 0.7)	0.6 (0.6, 0.7)
epiToc (1st)	0.1 (0.1, 0.2)	0.1 (0.1, 0.2)	0.2 (0.1, 0.2)	0.1 (0.1, 0.2)	0.1 (0.1, 0.2)	0.2 (0.1, 0.2)	0.2 (0.1, 0.2)	0.1 (0.1, 0.2)	0.1 (0.1, 0.2)	0.1 (0.1, 0.2)	0.1 (0.1, 0.2)	0.2 (0.1, 0.2)	0.2 (0.1, 0.2)	0.1 (0.1, 0.2)
Zhang Age (1st)	0.8 (0.8, 0.8)	0.8 (0.8, 0.8)	0.8 (0.8, 0.8)	0.8 (0.8, 0.8)	0.8 (0.8, 0.8)	0.8 (0.8, 0.8)	0.8 (0.7, 0.8)	0.8 (0.8, 0.8)	0.8 (0.8, 0.8)	0.8 (0.8, 0.8)	0.8 (0.8, 0.8)	0.8 (0.8, 0.8)	0.8 (0.7, 0.8)	0.8 (0.8, 0.8)
MiAge (1st)	0.1 (0.1, 0.2)	0.1 (0.1, 0.2)	0.1 (0.1, 0.2)	0.1 (0.1, 0.2)	0.1 (0.1, 0.2)	0.1 (0.1, 0.2)	0.1 (0.1, 0.2)	0.1 (0.1, 0.2)	0.1 (0.1, 0.2)	0.1 (0.1, 0.2)	0.1 (0.1, 0.2)	0.1 (0.1, 0.2)	0.1 (0.1, 0.2)	0.1 (0.1, 0.2)
DNAmTL (1st)	0.3 (0.3, 0.4)	0.4 (0.3, 0.4)	0.3 (0.3, 0.4)	0.3 (0.3, 0.4)	0.3 (0.3, 0.4)	0.3 (0.3, 0.4)	0.3 (0.3, 0.4)	0.4 (0.3, 0.4)	0.4 (0.3, 0.4)	0.3 (0.3, 0.4)	0.4 (0.3, 0.4)	0.4 (0.3, 0.4)	0.3 (0.3, 0.4)	0.3 (0.3, 0.4)
Zhang Mortality (2nd)	0.0 (0.0, 0.1)	0.1 (0.0, 0.1)	0.0 (0.0, 0.1)	0.0 (0.0, 0.1)	0.0 (0.0, 0.1)	0.0 (0.0, 0.1)	0.0 (0.0, 0.1)	0.0 (0.0, 0.1)	0.0 (0.0, 0.1)	0.0 (0.0, 0.1)	0.0 (0.0, 0.1)	0.0 (0.0, 0.1)	0.0 (0.0, 0.1)	0.0 (0.0, 0.1)
PhenoAge (2nd)	0.6 (0.6, 0.7)	0.6 (0.6, 0.7)	0.6 (0.6, 0.7)	0.6 (0.6, 0.7)	0.6 (0.6, 0.7)	0.6 (0.6, 0.7)	0.6 (0.6, 0.7)	0.6 (0.5, 0.7)	0.6 (0.6, 0.7)	0.6 (0.6, 0.7)	0.6 (0.6, 0.7)	0.6 (0.6, 0.7)	0.6 (0.5, 0.6)	0.6 (0.6, 0.7)
DunedinPoAm (2nd)	-0.1 (-0.1, 0.0)	-0.1 (-0.1, 0.0)	-0.1 (-0.1, 0.0)	-0.1 (-0.1, 0.0)	-0.1 (-0.1, 0.0)	-0.1 (-0.1, 0.0)	-0.1 (-0.1, 0.0)	0.0 (-0.1, 0.0)	-0.1 (-0.1, 0.0)	-0.1 (-0.1, 0.0)	-0.1 (-0.1, 0.0)	-0.1 (-0.1, 0.0)	-0.1 (-0.1, 0.0)	-0.1 (-0.1, 0.0)
GrimAge (2nd)	0.8 (0.7, 0.8)	0.8 (0.7, 0.8)	0.8 (0.7, 0.8)	0.8 (0.7, 0.8)	0.8 (0.7, 0.8)	0.7 (0.7, 0.8)	0.7 (0.7, 0.8)	0.8 (0.7, 0.8)	0.8 (0.7, 0.8)	0.7 (0.7, 0.8)	0.8 (0.7, 0.8)	0.8 (0.7, 0.8)	0.7 (0.7, 0.8)	0.7 (0.7, 0.8)

© 2024 Krieger N et al. *JAMA Network Open.*

E. Standardized Estimates (Unadjusted*) of Epigenetic Age Acceleration Associated With Body Mass Index (BMI), MBMS, Estimate and 95% CIs

Clock	Born in a Jim Crow State	Reversed ICE for Racialized Segregation at City of Birth (Black vs. White)	State Policy Conservatism in State of Birth	Parent's Highest Education	Participant's Highest Education	Experiences of Discrimination	Negative log10(household income / poverty line) in 2010 Dollars	Occupational Class	Housing Tenure — Paying Mortgage	Black Carbon	Pollution Proximity Index	Reversed Residential Census Tract ICE for Income	Reversed Residential Census Tract ICE for Racialized Economic Segregation	Reversed Residential Census Tract ICE for Racial Segregation	Reversed Residential Census Tract ICE for Housing Tenure
Horvath (1st)	0.1 (0.0, 0.2)	0.1 (0.0, 0.2)	0.1 (0.0, 0.2)	0.1 (-0.1, 0.2)	0.1 (0.0, 0.2)	0.1 (0.0, 0.2)	0.1 (0.0, 0.2)	0.1 (0.0, 0.2)	0.1 (0.0, 0.2)	0.1 (0.0, 0.2)	0.1 (0.0, 0.2)	0.1 (0.0, 0.2)	0.1 (0.0, 0.2)	0.1 (0.0, 0.2)	0.1 (0.0, 0.2)
Hannum (1st)	0.0 (-0.1, 0.1)	0.0 (-0.1, 0.1)	0.0 (-0.1, 0.1)	0.1 (0.0, 0.2)	0.0 (-0.1, 0.1)	0.0 (-0.1, 0.1)	0.0 (-0.1, 0.1)	0.0 (-0.1, 0.1)	0.0 (-0.1, 0.1)	0.0 (-0.1, 0.1)	0.0 (-0.1, 0.1)	0.0 (-0.1, 0.1)	0.0 (-0.1, 0.1)	0.0 (-0.1, 0.1)	0.0 (-0.1, 0.1)
epiToc (1st)	0.1 (0.0, 0.1)	0.1 (0.0, 0.1)	0.1 (0.0, 0.1)	0.1 (0.0, 0.2)	0.1 (0.0, 0.1)	0.1 (0.0, 0.1)	0.1 (0.0, 0.1)	0.1 (0.0, 0.1)	0.1 (0.0, 0.1)	0.1 (0.0, 0.1)	0.1 (0.0, 0.1)	0.1 (0.0, 0.1)	0.1 (0.0, 0.1)	0.1 (0.0, 0.1)	0.1 (0.0, 0.1)
Zhang Age (1st)	0.0 (-0.1, 0.0)	0.0 (-0.1, 0.0)	0.0 (-0.1, 0.0)	0.0 (-0.1, 0.1)	0.0 (-0.1, 0.0)	0.0 (-0.1, 0.0)	0.0 (-0.1, 0.0)	0.0 (-0.1, 0.1)	0.0 (-0.1, 0.0)	0.0 (-0.1, 0.0)	0.0 (-0.1, 0.0)	0.0 (-0.1, 0.1)	0.0 (-0.1, 0.1)	0.0 (-0.1, 0.0)	0.0 (-0.1, 0.1)
MiAge (1st)	0.1 (0.0, 0.1)	0.1 (0.0, 0.1)	0.1 (0.0, 0.1)	0.0 (-0.1, 0.1)	0.1 (0.0, 0.1)	0.1 (0.0, 0.1)	0.1 (0.0, 0.1)	0.1 (0.0, 0.1)	0.1 (0.0, 0.1)	0.1 (0.0, 0.1)	0.1 (0.0, 0.1)	0.1 (0.0, 0.2)	0.1 (0.0, 0.2)	0.1 (0.0, 0.1)	0.1 (0.0, 0.2)
DNAmTL (1st)	-0.1 (-0.2, 0.0)	-0.1 (-0.2, 0.0)	-0.1 (-0.2, 0.0)	-0.2 (-0.3, -0.1)	-0.2 (-0.2, -0.1)	-0.1 (-0.2, 0.0)	-0.1 (-0.2, 0.0)	-0.1 (-0.2, 0.0)	-0.1 (-0.2, 0.0)	-0.1 (-0.2, 0.0)	-0.1 (-0.2, 0.0)	-0.1 (-0.2, 0.0)	-0.1 (-0.2, 0.0)	-0.1 (-0.2, 0.0)	-0.1 (-0.2, 0.0)
Zhang Mortality (2nd)	0.0 (-0.1, 0.1)	0.0 (-0.1, 0.1)	0.0 (-0.1, 0.1)	0.0 (-0.1, 0.1)	0.0 (-0.1, 0.1)	0.0 (-0.1, 0.1)	0.0 (-0.1, 0.1)	0.0 (-0.1, 0.1)	0.0 (-0.1, 0.1)	0.0 (-0.1, 0.1)	0.0 (-0.1, 0.1)	0.0 (-0.1, 0.1)	0.0 (-0.1, 0.1)	0.0 (-0.1, 0.1)	0.0 (-0.1, 0.1)
PhenoAge (2nd)	0.1 (0.0, 0.1)	0.1 (0.0, 0.2)	0.1 (0.0, 0.2)	0.1 (0.0, 0.2)	0.1 (0.0, 0.2)	0.1 (0.0, 0.1)	0.1 (0.0, 0.2)	0.1 (0.0, 0.2)	0.1 (0.0, 0.2)	0.1 (0.0, 0.2)	0.1 (0.0, 0.2)	0.1 (0.0, 0.2)	0.1 (0.0, 0.2)	0.1 (0.0, 0.2)	0.1 (0.0, 0.2)
DunedinPoAm (2nd)	0.0 (-0.1, 0.1)	0.0 (-0.1, 0.1)	0.0 (-0.1, 0.1)	0.0 (-0.1, 0.1)	0.0 (-0.1, 0.1)	0.0 (-0.1, 0.1)	0.0 (-0.1, 0.1)	0.0 (-0.1, 0.1)	0.0 (-0.1, 0.1)	0.0 (-0.1, 0.1)	0.0 (-0.1, 0.1)	0.0 (-0.1, 0.1)	0.0 (-0.1, 0.1)	0.0 (-0.1, 0.1)	0.0 (-0.1, 0.1)
GrimAge (2nd)	0.0 (-0.1, 0.1)	0.0 (-0.1, 0.1)	0.0 (-0.1, 0.1)	0.0 (-0.1, 0.1)	0.0 (-0.1, 0.1)	0.0 (-0.1, 0.1)	0.0 (-0.1, 0.1)	0.0 (-0.1, 0.1)	0.0 (-0.1, 0.1)	0.0 (-0.1, 0.1)	0.0 (-0.1, 0.0)	0.0 (-0.1, 0.1)	0.0 (-0.1, 0.1)	0.0 (-0.1, 0.1)	0.0 (-0.1, 0.1)

F. Standardized Estimates (Unadjusted*) of Epigenetic Age Acceleration Associated With Body Mass Index (BMI), MESA US-Born, Estimate and 95% CIs

Clock	Born in a Jim Crow State	State Policy Conservatism in State of Birth	Parent's Highest Education	Participant's Highest Education	Major Discrimination Scale	Negative log10(household income / poverty line) in 2010 Dollars	Occupational Class	Housing Tenure — Paying Mortgage	Light Absorption Coefficient	Nitrous Oxides	Reversed Residential Census Tract ICE for Income	Reversed Residential Census Tract ICE for Racialized Economic Segregation	Reversed Residential Census Tract ICE for Racial Segregation	Reversed Residential Census Tract ICE for Housing Tenure
Horvath (1st)	0.1 (0.0, 0.1)	0.0 (0.0, 0.1)	0.1 (0.0, 0.1)	0.1 (0.0, 0.1)	0.1 (0.0, 0.1)	0.1 (0.0, 0.1)	0.0 (0.0, 0.1)	0.0 (0.0, 0.1)	0.0 (0.0, 0.1)	0.0 (0.0, 0.1)	0.1 (0.0, 0.1)	0.1 (0.0, 0.1)	0.1 (0.0, 0.1)	0.1 (0.0, 0.1)
Hannum (1st)	0.0 (0.0, 0.0)	0.0 (0.0, 0.0)	0.0 (0.0, 0.0)	0.0 (0.0, 0.0)	0.0 (0.0, 0.0)	0.0 (0.0, 0.0)	0.0 (0.0, 0.0)	0.0 (0.0, 0.0)	0.0 (0.0, 0.0)	0.0 (0.0, 0.0)	0.0 (0.0, 0.0)	0.0 (0.0, 0.0)	0.0 (0.0, 0.0)	0.0 (0.0, 0.0)
epiToc (1st)	0.0 (-0.1, 0.0)	0.0 (-0.1, 0.0)	0.0 (-0.1, 0.0)	0.0 (-0.1, 0.0)	0.0 (-0.1, 0.0)	0.0 (-0.1, 0.0)	0.0 (-0.1, 0.0)	0.0 (-0.1, 0.0)	0.0 (-0.1, 0.0)	0.0 (-0.1, 0.0)	0.0 (-0.1, 0.0)	0.0 (-0.1, 0.0)	0.0 (-0.1, 0.0)	0.0 (-0.1, 0.0)
Zhang Age (1st)	0.0 (0.0, 0.0)	0.0 (0.0, 0.0)	0.0 (0.0, 0.0)	0.0 (0.0, 0.0)	0.0 (0.0, 0.0)	0.0 (0.0, 0.0)	0.0 (0.0, 0.0)	0.0 (0.0, 0.0)	0.0 (0.0, 0.0)	0.0 (0.0, 0.0)	0.0 (0.0, 0.0)	0.0 (0.0, 0.0)	0.0 (0.0, 0.0)	0.0 (0.0, 0.0)
MiAge (1st)	0.0 (-0.1, 0.0)	0.0 (-0.1, 0.0)	0.0 (-0.1, 0.0)	0.0 (-0.1, 0.0)	0.0 (-0.1, 0.0)	0.0 (-0.1, 0.0)	0.0 (-0.1, 0.0)	0.0 (-0.1, 0.0)	0.0 (-0.1, 0.0)	0.0 (-0.1, 0.0)	0.0 (-0.1, 0.0)	0.0 (-0.1, 0.0)	0.0 (-0.1, 0.0)	0.0 (-0.1, 0.0)
DNAmTL (1st)	0.0 (0.0, 0.1)	0.0 (0.0, 0.1)	0.0 (0.0, 0.1)	0.0 (0.0, 0.1)	0.0 (0.0, 0.1)	0.0 (0.0, 0.1)	0.0 (0.0, 0.1)	0.0 (0.0, 0.1)	0.0 (0.0, 0.1)	0.0 (0.0, 0.1)	0.0 (0.0, 0.1)	0.0 (0.0, 0.1)	0.0 (0.0, 0.1)	0.0 (0.0, 0.1)
Zhang Mortality (2nd)	0.0 (0.0, 0.1)	0.0 (0.0, 0.1)	0.0 (0.0, 0.1)	0.0 (0.0, 0.1)	0.0 (0.0, 0.1)	0.0 (0.0, 0.1)	0.0 (0.0, 0.1)	0.0 (0.0, 0.1)	0.0 (0.0, 0.1)	0.0 (0.0, 0.1)	0.0 (0.0, 0.1)	0.0 (0.0, 0.1)	0.0 (0.0, 0.1)	0.0 (0.0, 0.1)
PhenoAge (2nd)	0.1 (0.0, 0.1)	0.1 (0.0, 0.1)	0.1 (0.0, 0.1)	0.1 (0.0, 0.1)	0.1 (0.0, 0.1)	0.1 (0.0, 0.1)	0.1 (0.0, 0.1)	0.1 (0.0, 0.1)	0.1 (0.0, 0.1)	0.1 (0.0, 0.1)	0.1 (0.0, 0.1)	0.1 (0.0, 0.1)	0.1 (0.0, 0.1)	0.1 (0.0, 0.1)
DunedinPoAm (2nd)	0.1 (0.0, 0.1)	0.1 (0.0, 0.1)	0.1 (0.0, 0.1)	0.1 (0.0, 0.1)	0.1 (0.0, 0.1)	0.1 (0.0, 0.1)	0.1 (0.0, 0.1)	0.1 (0.0, 0.1)	0.1 (0.0, 0.1)	0.1 (0.0, 0.1)	0.1 (0.0, 0.1)	0.1 (0.0, 0.1)	0.1 (0.0, 0.1)	0.1 (0.0, 0.1)
GrimAge (2nd)	0.0 (0.0, 0.1)	0.0 (0.0, 0.1)	0.0 (0.0, 0.1)	0.0 (0.0, 0.1)	0.0 (0.0, 0.1)	0.0 (0.0, 0.1)	0.0 (0.0, 0.1)	0.0 (0.0, 0.1)	0.0 (0.0, 0.1)	0.0 (0.0, 0.1)	0.0 (0.0, 0.1)	0.0 (0.0, 0.1)	0.0 (0.0, 0.1)	0.0 (0.0, 0.1)

* unadjusted for multiple comparisons

© 2024 Krieger N et al. *JAMA Network Open.*

eAppendix 1. Construction of Surrogate Variables and Genetic Principal Component (PC) Variables

Surrogate variables: We adjusted for batch effects using surrogate variables (SVs) (the first 5 for MBMS and the first 10 for MESA, calculated using the R package sva [1]). We used SVs to adjust for batch because stratifying the dataset meant that measured batch variables had small cell frequencies, which in turn caused uncertainty in the regression model and resulted in deflation of test statistics. We chose to use 5 SVs for MBMS and 10 for MESA as these encompassed the majority of association between SVs and batch variables, as well as DNA input level for MBMS; and their inclusion returned EWAS lambda values to approximately 1.

[1] Leek JT, Johnson WE, Parker HS, Fertig EJ, Jaffe AE, Zhang Y, Storey JD, Torres LC (2023). *sva: Surrogate Variable Analysis*. R package version 3.48.0.

Genetic principal component (PC) variables: MESA used the Affymetrix Genome-Wide Human SNP Array 6.0 to genotype 8402 participants; this overlaps with 933 out of 975 of the US-born participants with DNAm data. Genetic principal components were generated by MESA: 23,428 flagged SNPs and 6,849 SNPs in long range LD were removed before principal components were computed per chromosome, and then combined across chromosomes to give the final PC values. We used the genetic PCs provided to us by MESA that were estimated in all participants combined, as all participants were included together in the models.

Of note, we employed the SVs in the primary analyses because these could be used in both the MBMS and MESA analyses; by contrast, the genetic PCs could be employed solely with the MESA data.

© 2024 Krieger N et al. *JAMA Network Open.*

eAppendix 2. Definitions and Sources of Metrics Included in Table 1

1) MBMS inclusion criteria were that all participants had to self-identify as being US-born and being either Black non-Hispanic or white non-Hispanic; MESA did not have these nativity restrictions, and in the Exam 5 epigenetic subsample, 77.1% were US-born and 22.7% were born outside of the US, such that data on US city of birth are N/A (N/A). Additionally, although the surveys had many identical or similar questions, in some cases some questions were unique to one survey, in which case the data are listed as "N/A" for the other survey.

2) We report on the data only of the US-born MESA participants, excluding all foreign-born participants and the two MESA participants who did not specify their country or state of birth.

3) We use the racial/ethnic and sex/gender terminology employed in each study in the self-report questions asked of each participant, and do not have data as to whether participants identified as cisgender or transgender.

4) We computed the Index of Concentration at the Extreme for race/ethnicity for the MBMS participants' city of birth in relation to the 1940, 1950, and 1960 census for the categories "White" versus "Negro" and interpolated the values and used the value for the birth year of the participant; MESA collected data only on state, not city, of birth

5) The US state policy liberalism index, for state of birth at time of birth, is based on application of a dynamic latent-variable model to data on 148 policies collected over eight decades (1936-2014); the source is: Caughey D, Warshaw CS. Data for participants whose birth years were prior to the years modeled in states except for Alaska and Hawaii were assigned the 10-year average of the liberalism index for their birth states' first 10 years modeled. Estimates for the District of Columbia state policy liberalism index were not available. The dynamics of state policy liberalism, 1936-2014. *Am J Political Science* 2016; 60(4):899-913. URI: http://hdl.handle.net/1721.1/105870

6) Explicit self-report measures of racial discrimination

-- MBMS used the Experiences of Discrimination scale (EOD), which explicitly asks about experiences of racial discrimination in the 9 specified domains (at school; getting hired or getting a job; at work; getting housing; getting medical care; getting service in a store or restaurant; getting credit, bank loans, or a mortgage; on the street or in a public setting; from the police or in the courts) [sources: Krieger N, Smith K, Naishadham D, Hartman C, Barbeau EM. Experiences of discrimination: validity and reliability of a self-report measure for population health research on racism and health. *Soc Sci Med.* 2005 Oct; 61(7):1576-96; Krieger N, Waterman PD, Kosheleva A, Chen JT, Carney DR, Smith KW, Bennett GG, Williams DR, Freeman E, Russell B, Thornhill G, Mikolowsky K, Rifkin R, Samuel L. Exposing racial discrimination: implicit & explicit measures--the My Body, My Story study of 1005 US-born black & white community health center members. *PLoS One*. 2011; 6(11):e27636].

-- MESA asked analogous questions about self-reported experiences of discrimination in relation to specified domains only in Exam 1 (2000-2002), i.e., 10 years earlier than Exam 5, for which they employed the Major Discrimination Scale (MDS), which asks about experiences of discrimination (ever) in 6 domains (unfairly fired or denied promotion; unfairly not hired; stopped, searched, questioned, physically threatened or abused by the police; discouraged by a teacher or advisor from continuing your education; prevented from moving into a neighborhood by landlord or realtor; moved into neighborhood where neighbors made life difficult for your family) and for each question asked people to choose the "main" reason for this unfair treatment (race or ethnicity; gender; age; religion; physical appearance; sexual orientation; income level/social class; other) [sources: Williams DR, Yan Yu, Jackson JS, Anderson NB. Racial Differences in Physical and Mental Health: Socio-economic Status, Stress and Discrimination. *J Health Psychol.* 1997 Jul;2(3):335-51; Borrell LN, Diez Roux AV, Jacobs DR Jr, Shea S, Jackson SA, Shrager S, Blumenthal RS. Perceived racial/ethnic discrimination, smoking and alcohol consumption in the Multi-Ethnic Study of Atherosclerosis (MESA). *Prev Med*. 2010 Sep-Oct;51(3-4):307-12]. For the MDS metric employed in our study, we computed the N of domains in which the participants reported having experienced the unfair treatment and saying the main reason for that experience was race/ethnicity.

7) Income data are adjusted from their survey year to 2010 US Dollars using the Consumer Price Index data from the US Bureau of Labor, see CPI Inflation Calculator https://www.bls.gov/data/inflation_calculator.htm ; (accessed November 18, 2021).

8) This ratio is computed using the US poverty threshold data (by household size and age composition) for the year in which the survey was administered; see: https://www.census.gov/data/tables/time-series/demo/income-poverty/historical-poverty-thresholds.html (accessed November 14, 2021)

9) For black carbon, the MBMS measure reflects model estimates of average annual black carbon in the atmosphere at the participants' residential address for the year prior to their survey data (see: Krieger N, Waterman PD, Gryparis A, Coull BA. Black carbon exposure, socioeconomic and racial/ethnic spatial polarization, and the Index of Concentration at the Extremes (ICE). *Health & Place* 2015; 34:215-228; and Gryparis A, Coull BA, Schwartz J, Suh HH. Semiparametric latent variable regression models for spatiotemporal modeling of mobile source particles in the greater Boston area. *Appl Statist.* 2007;56:183–209).

For MBMS, the Pollution Proximity Index data reflect scores from 0-5 where scores are assigned based on the quintile ranges of emissions intensity values across six pollutants (CO, SO_2, NO_2, NO_x, $PM_{2.5}$ and CO_2) for the year 2012, see: https://www.mapc.org/wp-content/uploads/2020/05/PPA_Technical_Memorandum.pdf (accessed November 14, 2021). For MESA, the light absorption coefficients reflect a model estimated average of 2-week predictions from January, 2000, to December, 2010, generated at each participant's baseline address. For the light absorption coefficient, 0.5×10^{-5}/m is approximately equivalent to 0.5 μg/m³ of black carbon. See, for MESA: Kaufman JD, Spalt EW, Curl CL, Hajat A, Jones MR, Kim SY, Vedal S, Szpiro AA, Gassett A, Sheppard L, Daviglus ML, Adar SD. Advances in understanding air pollution and CVD. *Glob Heart* 2016; 11(3):343-352 .

10) For the ICE for racialized economic segregation, high-income refers to the top quintile for US household income and low-income refers to the bottom quintile for US household income, during the years specified; for further explication, see: https://www.hsph.harvard.edu/thegeocodingproject/covid-19-resources/ (accessed November 14, 2021); see also: Krieger N, Waterman PD, Gryparis A, Coull BA. Black carbon exposure, socioeconomic and racial/ethnic spatial polarization, and the Index of Concentration at the Extremes (ICE). *Health & Place* 2015; 34:215-228; and Krieger N, Feldman JM, Waterman PD, Chen JT, Coull BA, Hemenway D. Local residential segregation matters: stronger association of census tract compared to conventional city-level measures with fatal and non-fatal assaults (total and firearm related), using the Index of Concentration at the Extremes (ICE) for racial, economic, and racialized economic segregation, Massachusetts (US), 1995-2010. *J Urban Health* 2017; 94:244-258.

11) Body mass index was calculated from height and weight collected at study-time for participants in MBMS and MESA. BMI was calculated as weight in kilograms divided by height in meters squared (kg/m^2).

© 2024 Krieger N et al. *JAMA Network Open.*

eAppendix 3. Methods for Multiple Imputation and Multiple Comparisons

1) Multiple imputation

Multiple imputation was carried out in all models to account for missing exposures and covariates. The extent of missing data on each of the variables is listed in Table 1. The imputation models included the epigenetic clock values, age, racialized group, each exposure interacted with racialized groups, BMI, smoking, sex/gender, the cell type variables, and the surrogate variables (5 for MBMS and 10 for MESA). All analytic variables were included in the imputation models andiInteractions were included to reduce the potential for bias. The default, predictive mean matching, was used as the imputation method for each missing variable and 40 imputations were carried out per model. Multiple imputations were performed using the mice package in R.

References:

Buuren S, Groothuis-Oudshoorn K. mice: Multivariate Imputation by Chained Equations in R. *J Statistical Software* 2011; 45(3):1-67.

Tilling K, Williamson EJ, Spratt M, Sterne JAC, Carpenter JR. Appropriate inclusion of interactions was needed to avoid bias in multiple imputation. *J Clin Epidemiol* 2016; 80:107–115.

2) Multiple comparisons

The number of multiple comparisons varied and depended on four aspects of each set of analyses: (1) the set of variables considered (e.g., for early life or for adult life exposures), (2) which population group was the focus (MBMS or MESA), (3) the number of racialized groups at issue, and (4) whether the analyses focused on the epigenetic clocks individually or the pooled estimates. As a result, depending on the specific analysis at issue, the number of multiple comparisons ranged between 9 and 350. We list the N of multiple comparisons for each set of analyses below; further details (e.g., specific variables included) can be provided upon request.

**Analysis**	**N of multiple comparisons**
Individual clocks:	
Multiple comparisons N for MBMS early life exposures, individual clocks	90
Multiple comparisons N for MBMS adult life exposures, individual clocks	260
Multiple comparisons N for MESA early life exposures, individual clocks	120
Multiple comparisons N for MESA adult life exposures, individual clocks	350
Pooled clocks: each generation (1st and 2nd) pooled as its own clock	
(1st Generation Pooled and 2nd Generation Pooled [essentially 2 constructed new "clocks"]), MBMS, Early Life Exposures	18
(1st Generation Pooled and 2nd Generation Pooled), MBMS, Adult Life Exposures	52
(1st Generation Pooled and 2nd Generation Pooled), MESA, Early Life Exposures	24
(1st Generation Pooled and 2nd Generation Pooled), MESA, Adult Life Exposures	70
Pooled clocks: pooled across the full range of clocks (1st & 2nd generation)	
(1st and 2nd Generation Pooled [essentially 1 constructed new "clock"]), MBMS, Early Life Exposures	9
(1st and 2nd Generation Pooled), MBMS, Adult Life Exposures	26
(1st and 2nd Generation Pooled), MESA, Early Life Exposures	12
(1st and 2nd Generation Pooled), MESA, Adult Life Exposures	35

Overall significance level was set to 0.05, but results were only considered significant if the p-value of the model estimates were less than the calculated p-value threshold, which varied based on the number of comparisons.

© 2024 Krieger N et al. *JAMA Network Open.*

eFigure 1. Additional Variance Explained by Incorporating Genetic PCs

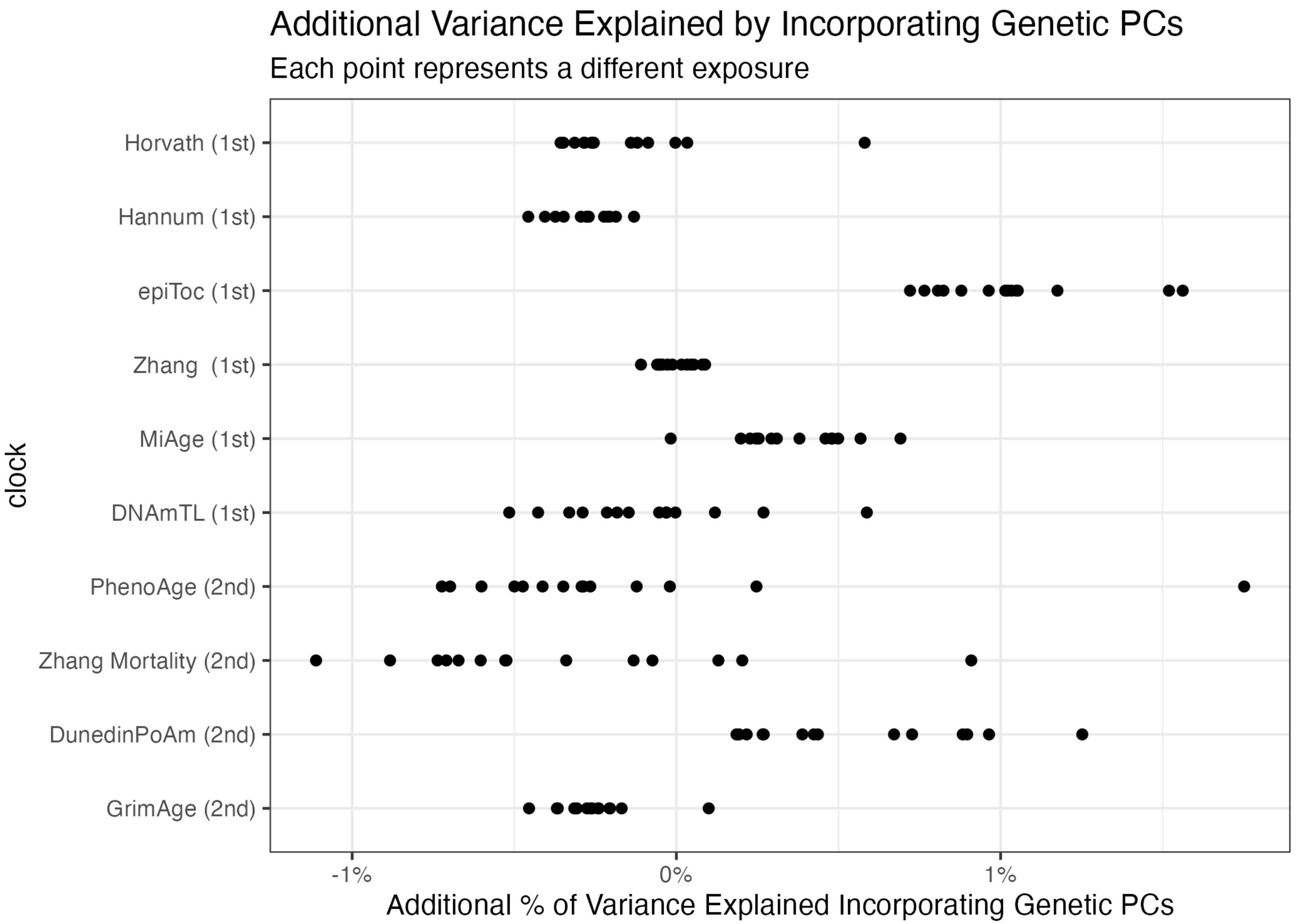

The average additional % variance explained by incorporating the genetic PCs was 0.06%, and the maximum was 1.75%. Decreases in variance explained can be explained by the SVs being determined by an iterative (stochastic) algorithm, and compared to using the SVs, the use of genetic PCs had no discernable impact on effect estimates.

© 2024 Krieger N et al. *JAMA Network Open.*

eFigure 2. Correlation of Chronological Age and Epigenetic Aging by Epigenetic Clock: MESA and MBMS

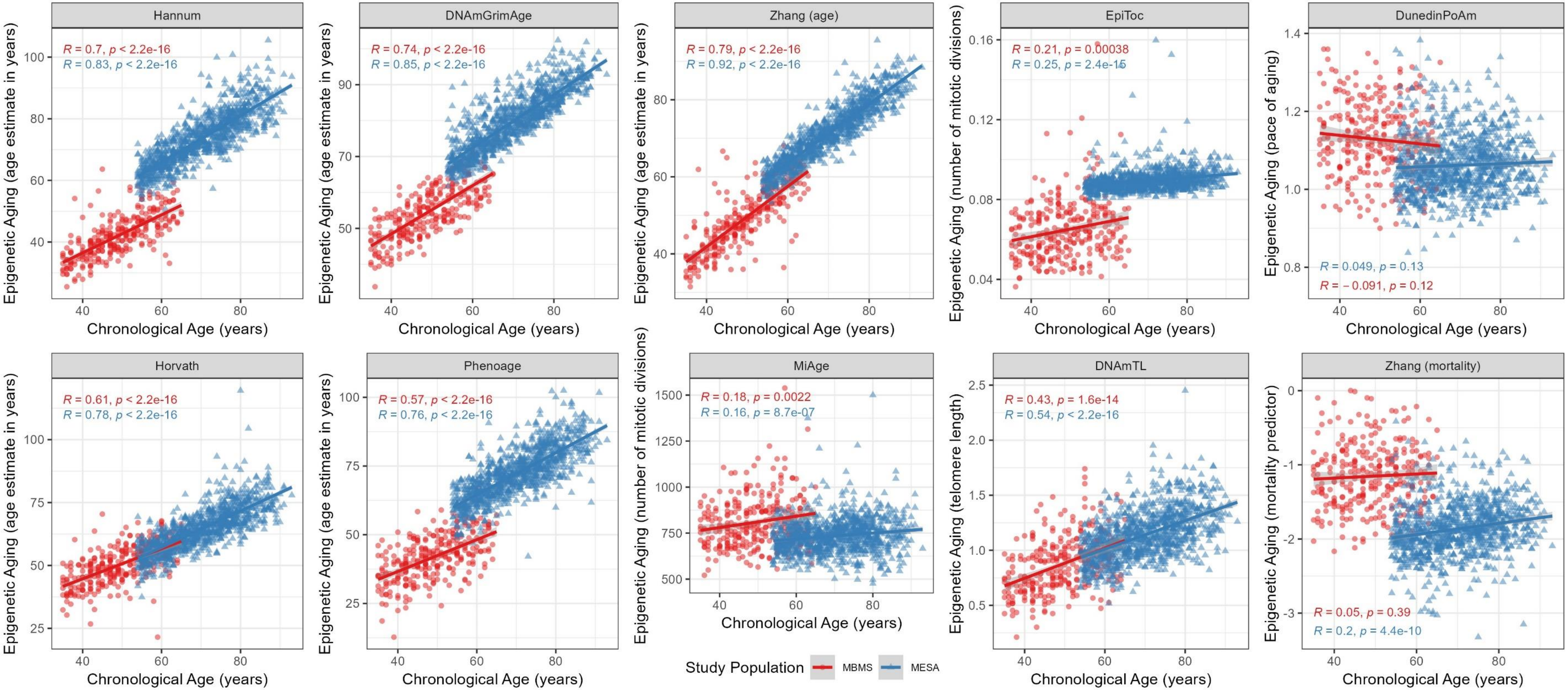

Note:
(1) Each scatterplot includes the Pearson correlation coefficient R and its p-value.
(2) Not all of the epigenetic clock measures are interpretable on the age (years) scale. For the ones that are (Horvath, Hannum, Zhang, PhenoAge, GrimAge), age acceleration (based on detrending the raw clock measures for chronological age) is interpretable as years of age acceleration (relative to chronological age). For the epigenetic clocks that are not on the years of age scale (EpiToc, MiAge, DNAmTL, Zhang mortality, and DunedinPoAm), interpretation is specific to the clock (e.g. EpiToc and MiAge are on the scale of cell mitotic divisions, Zhang is based on a predicted risk score for mortality, and DunedinPoAm is based on rate of change in biomarkers longitudinally). This information is summarized in eTable 1 and in the footnote to Figure 1.

© 2024 Krieger N et al. *JAMA Network Open.*

eFigure 3. Correlation Matrix for Continuous Variables From MBMS

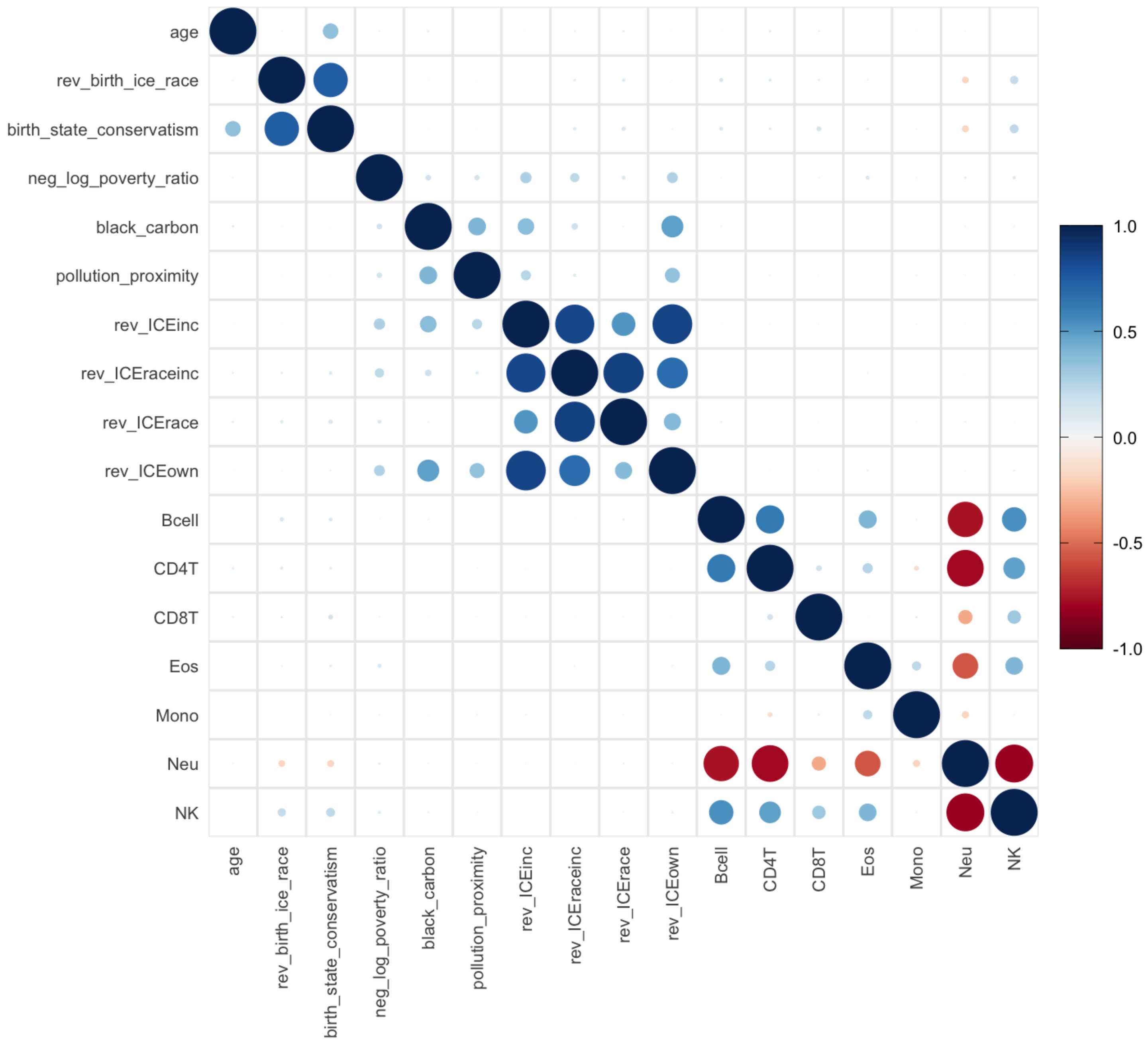

© 2024 Krieger N et al. *JAMA Network Open.*

eFigure 4. Correlation Matrix for Continuous Variables From MESA, US-Born

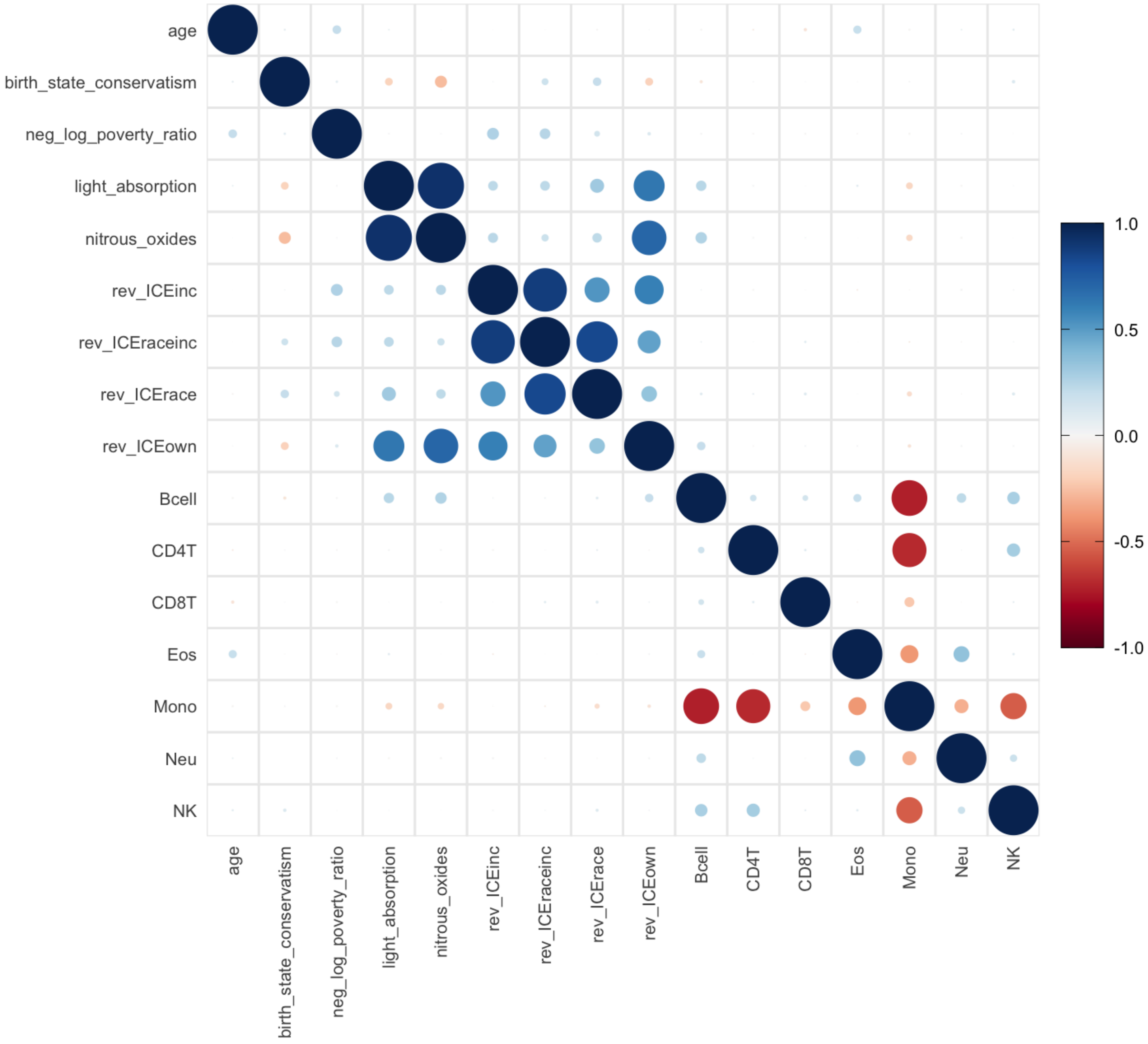

© 2024 Krieger N et al. *JAMA Network Open.*

eFigure 5. MBMS: Standardized Effect Estimates (Unadjusted) (95% CI) for Early Life Adverse Exposure and Adult Racial Discrimination

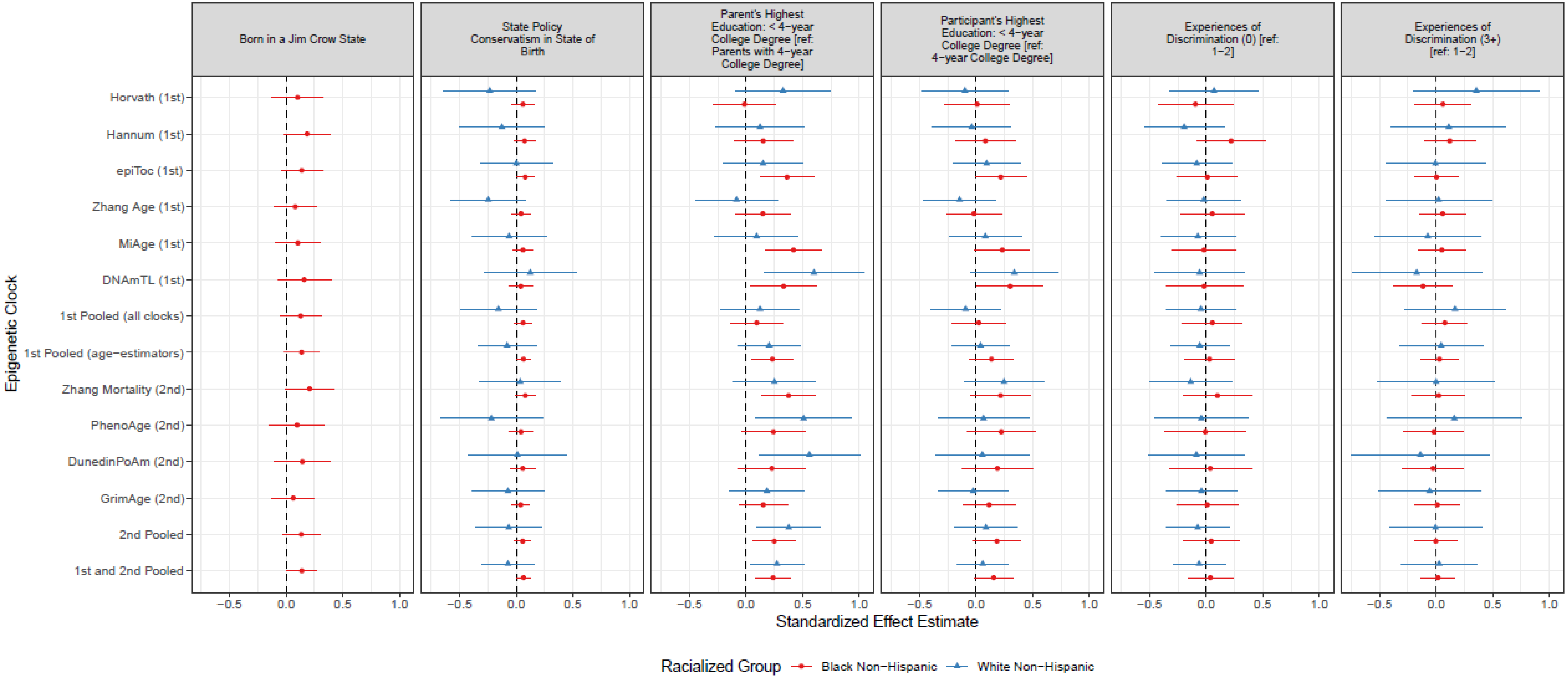

* unadjusted for multiple comparisons

© 2024 Krieger N et al. *JAMA Network Open.*

eFigure 6. MBMS: Standardized Effect Estimates (Unadjusted) (95% CI) for Adult Economic, Pollution, and Segregation Adverse Exposures

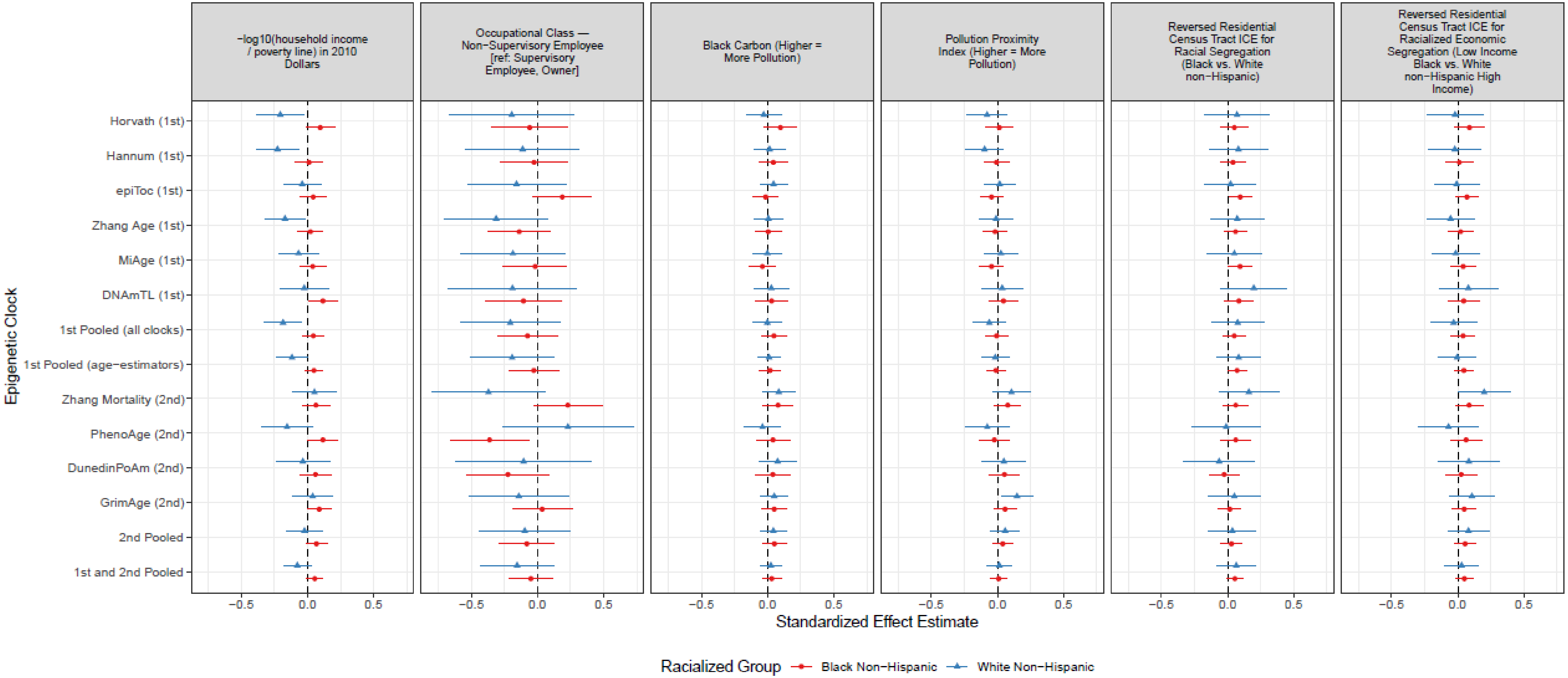

* unadjusted for multiple comparisons

© 2024 Krieger N et al. *JAMA Network Open.*

eFigure 7. MESA: Standardized Effect Estimates (Unadjusted) (95% CI) for Early Life Adverse Exposure and Adult Racial Discrimination

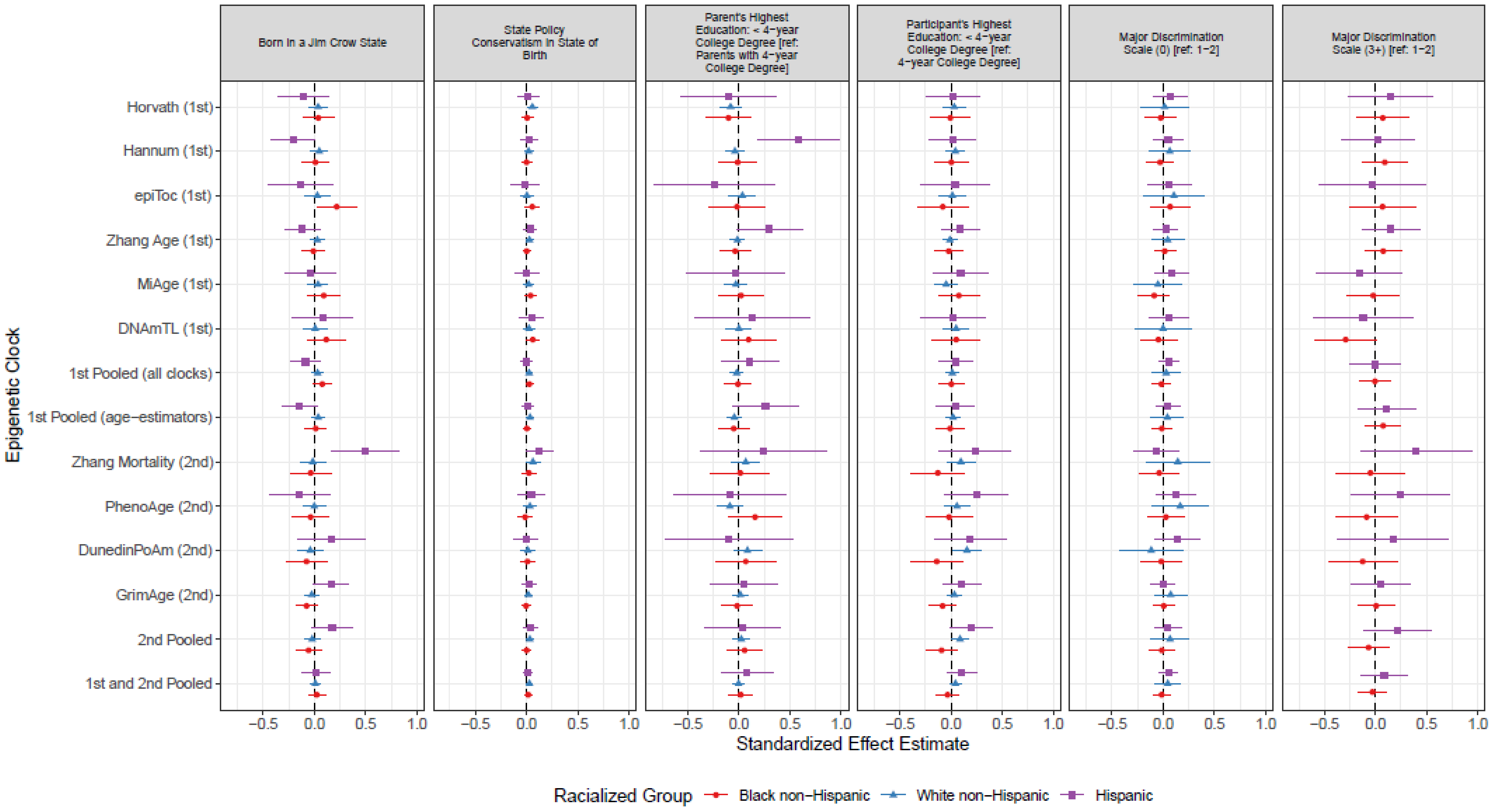

* unadjusted for multiple comparisons

© 2024 Krieger N et al. *JAMA Network Open.*

eFigure 8. MESA: Standardized Effect Estimates (Unadjusted) (95% CI) for Adult Economic, Pollution, and Segregation Adverse Exposures

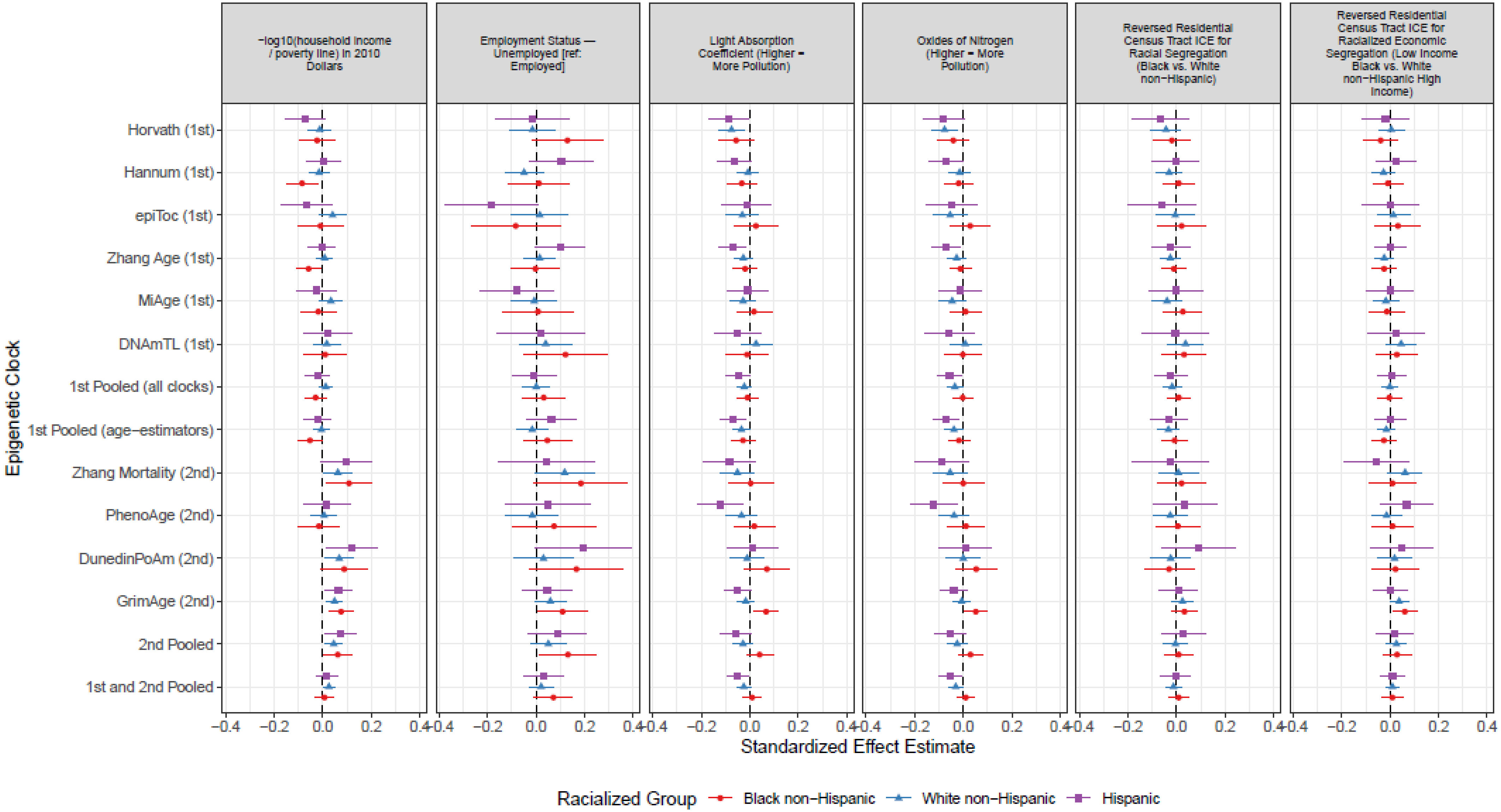

* unadjusted for multiple comparisons

© 2024 Krieger N et al. *JAMA Network Open.*

eFigure 9. Standardized Effect Estimates (Unadjusted*) and 95% CIs by Racialized Group for Early Life Adverse Exposures (Reversed Index of Concentration at the Extremes for Racialized Segregation at City of Birth) and Adult Individual-, Household-, and Area-Based Adverse Exposures (Employment Status; Housing Tenure, and Reversed Index of Concentration at the Extremes for Income and Housing Tenure): MBMS Participants

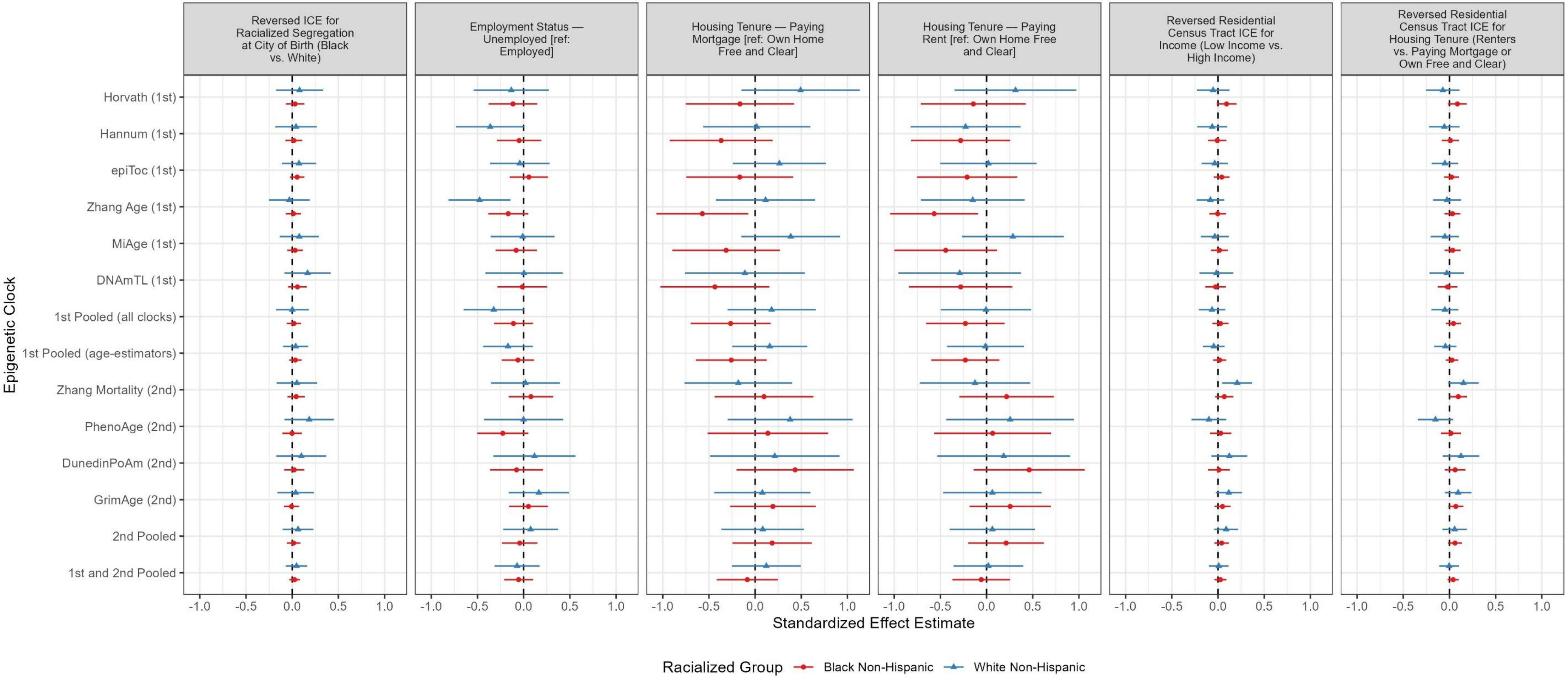

* unadjusted for multiple comparisons

© 2024 Krieger N et al. *JAMA Network Open.*

eFigure 10. Standardized Effect Estimates (Unadjusted) and 95% CIs by Racialized Group for Early Life Adverse Exposures (Reversed Index of Concentration at the Extremes for Racialized Segregation at City of Birth) and Adult Household- and Area-Based Adverse Exposures (Employment Status; Housing Tenure, and Reversed Index of Concentration at the Extremes for Income and Housing Tenure): MESA Participants

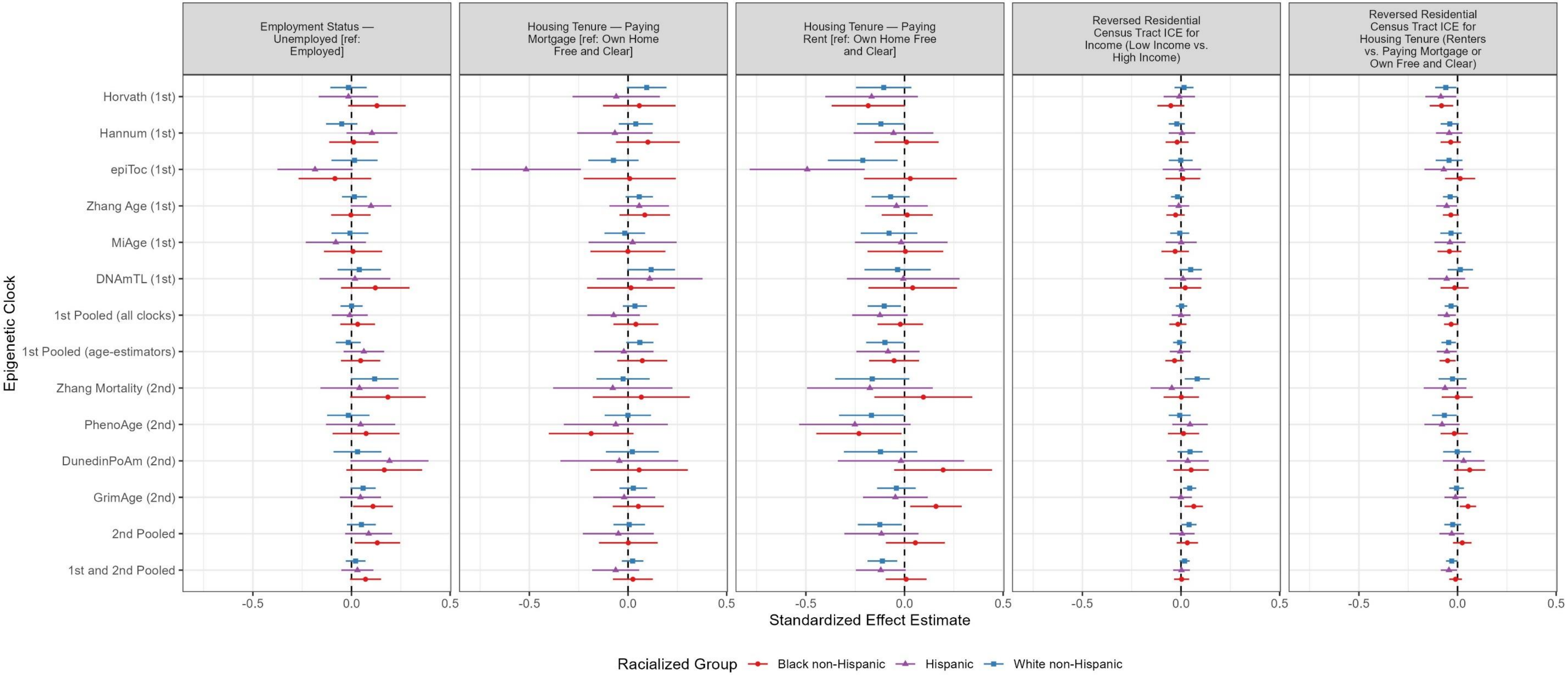

* unadjusted for multiple comparisons

© 2024 Krieger N et al. *JAMA Network Open.*

eFigure 11. Standardized Effect Estimates (Unadjusted) by Racialized Group and 95% CIs for Early Life Exposures (Born in a Jim Crow State, Reversed Index of Concentration at the Extremes for Racialized Segregation at City of Birth, State Policy Conservatism in State of Birth, Parents' Education, Participants' Education): MBMS, Not Including Covariate Data on BMI and Smoking

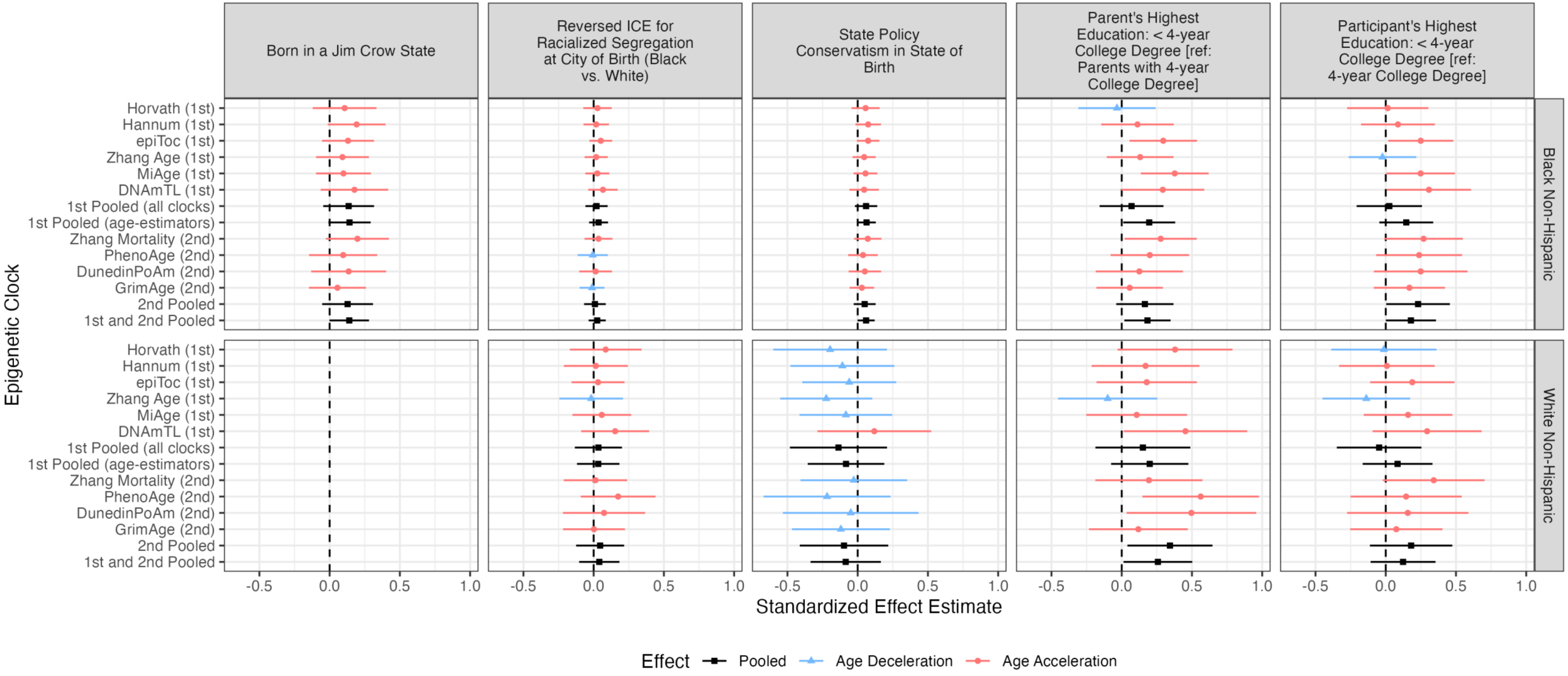

* unadjusted for multiple comparisons

The main analyses include smoking and BMI as they were hypothesized to be significantly explanatory with respect to the variation observed in the epigenetic clocks. However, it is possible that adult-age characteristics like smoking and BMI may be causally downstream of early life exposures and/or effect modifiers of contemporaneous adult life exposures. Therefore, both analyses including them (in the main figures) and analyses without them (eFigures S4-S9) are presented. Because only 2 of the MBMS white non-Hispanic participants were born in a Jim Crow state (see Table 1), we do not report results for this group, given small numbers.

© 2024 Krieger N et al. *JAMA Network Open.*

eFigure 12. Standardized Effect Estimates (Unadjusted) by Racialized Group and 95% CIs for Adult Life Exposures (Set 1: Experiences of Discrimination, Negative log Household Income Ratio to the Poverty Line, Occupational Class, Employment Status, Housing Tenure, and Black Carbon Air Pollution): MBMS, Not Including Covariate Data on BMI and Smoking

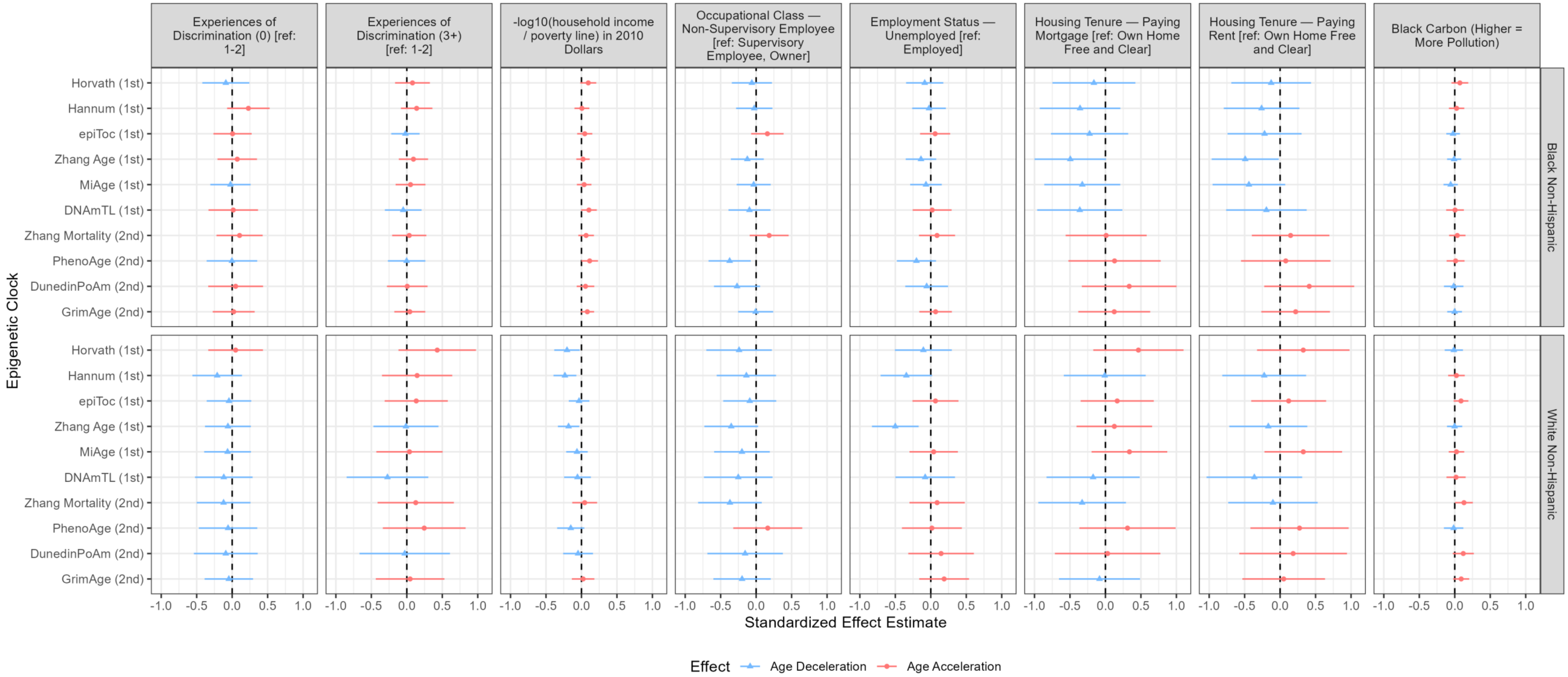

* unadjusted for multiple comparisons

© 2024 Krieger N et al. *JAMA Network Open.*

eFigure 13. Standardized Effect Estimates (Unadjusted) by Racialized Group and 95% CIs for Adult Life Exposures (Set 2: Pollution Proximity Index [Air Pollution], Reversed Index of Concentration at the Extremes for Income, Racial Segregation, Racialized Economic Segregation, and Housing Tenure): MBMS, Not Including Covariate Data on BMI and Smoking

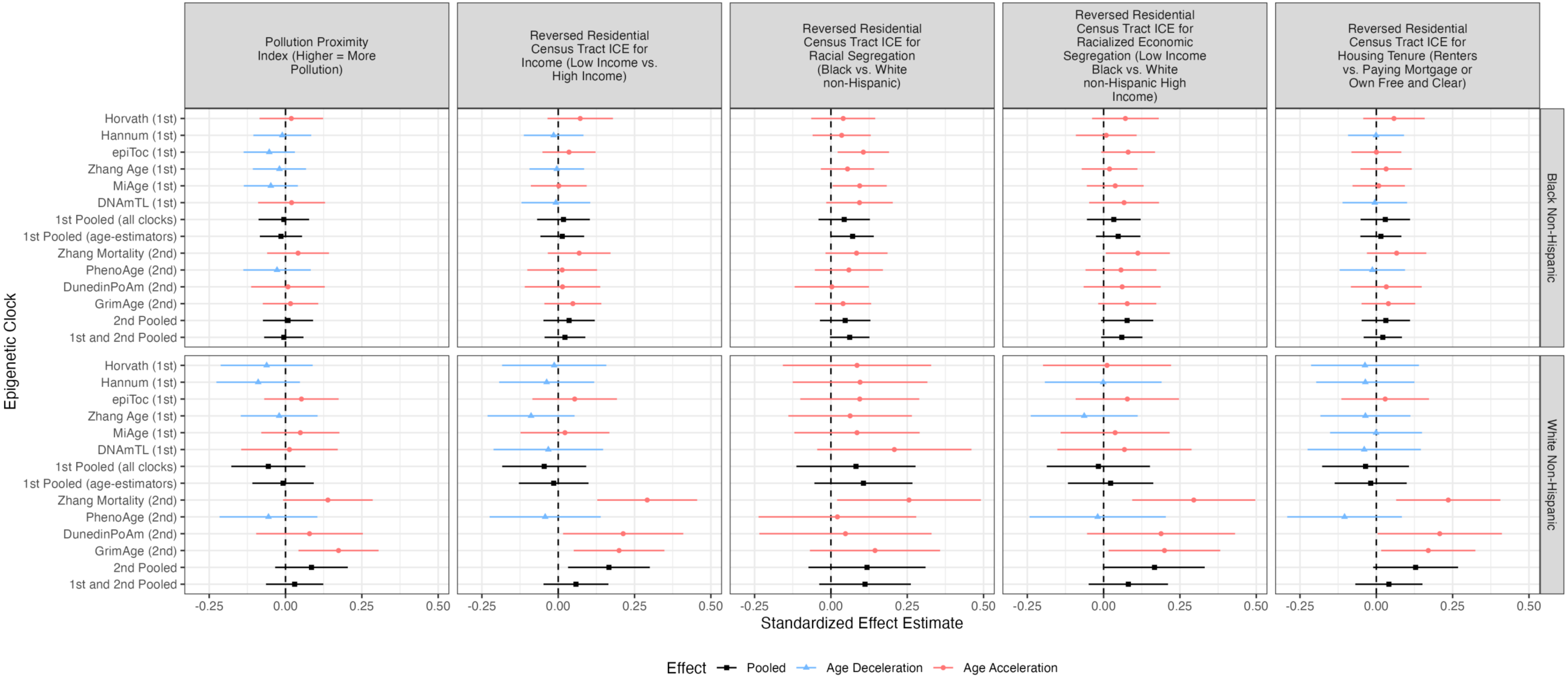

* unadjusted for multiple comparisons

© 2024 Krieger N et al. *JAMA Network Open.*

eFigure 14. Standardized Effect Estimates (Unadjusted) by Racialized Group and 95% CIs for Early Life Exposures (Born in a Jim Crow State, State Policy Conservatism in State of Birth, Parents' Education, Participants' Education): MESA US-Born, Not Including Covariate Data on BMI and Smoking

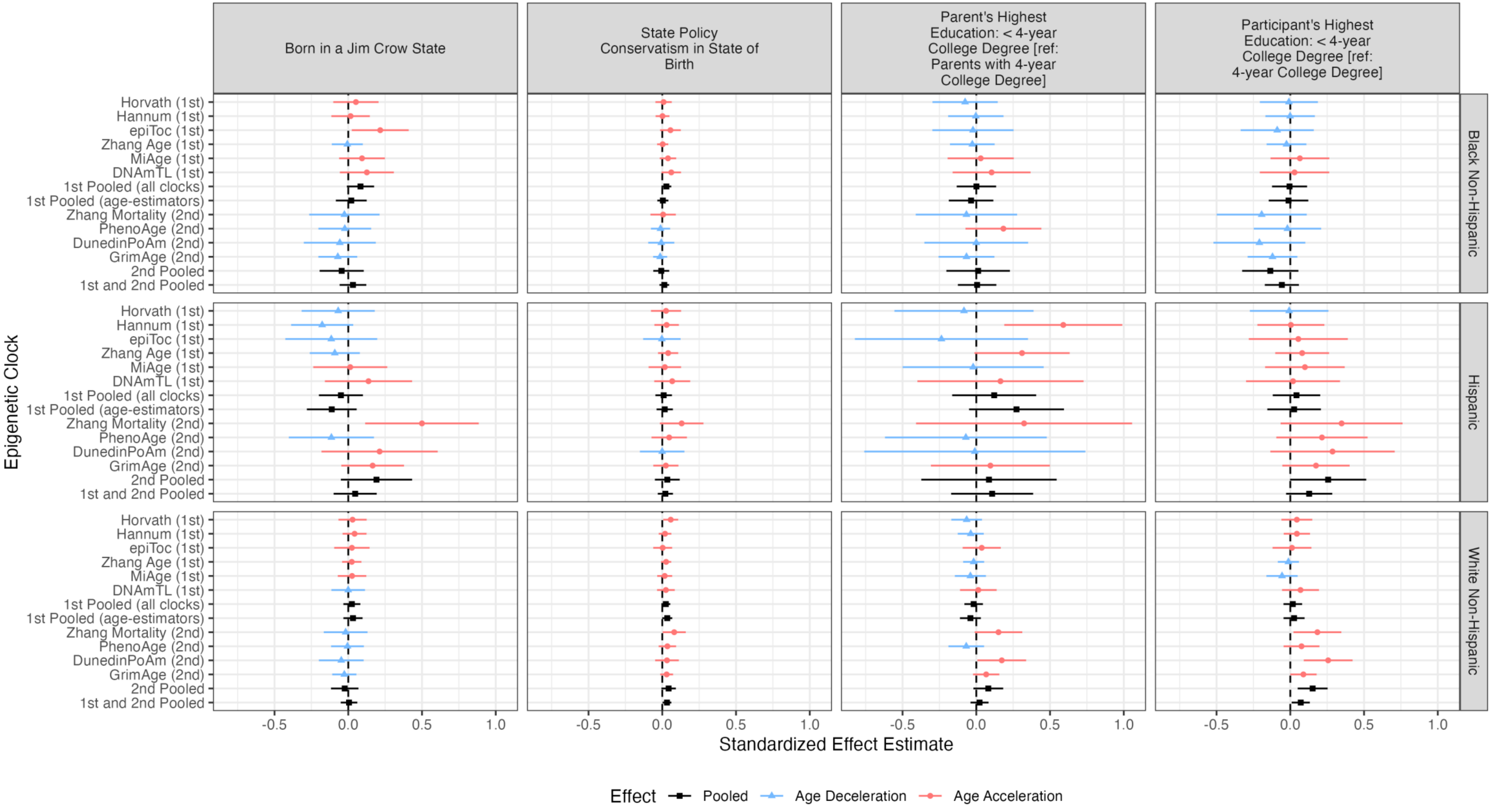

* unadjusted for multiple comparisons

© 2024 Krieger N et al. *JAMA Network Open.*

eFigure 15. Standardized Effect Estimates (Unadjusted) by Racialized Group and 95% CIs for Adult Life Exposures (Set 1: Major Discrimination Scale (racialized), Negative log Household Income Ratio to the Poverty Line, Employment Status, Housing Tenure, and Light Absorption Coefficient [Air Pollution]): MESA US-Born, Not Including Covariate Data on BMI and Smoking

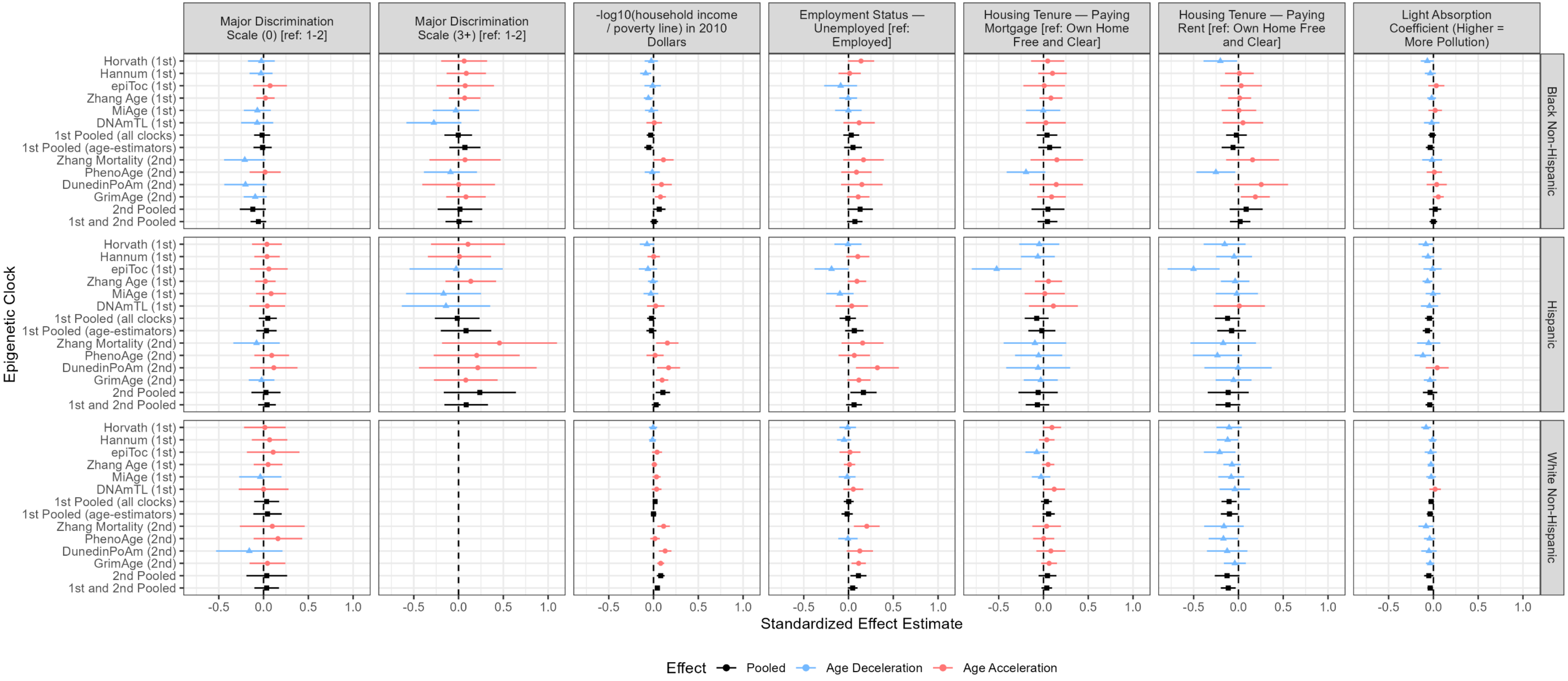

* unadjusted for multiple comparisons

For the Major Discrimination Score (referring to discrimination reported by participants who attributed their reported unfair experiences as due to their race/ethnicity), no MESA white non-Hispanic participants had score >=3 (see Table 1), so there are no data to report for this category.

© 2024 Krieger N et al. *JAMA Network Open.*

eFigure 16. Standardized Effect Estimates (Unadjusted) by Racialized Group and 95% CIs for Adult Life Exposures (Set 2: Oxides of Nitrogen [Air Pollution], Reversed Index of Concentration at the Extremes for: Income, Racial Segregation, Racialized Economic Segregation, and Housing Tenure): MESA US-Born, Not Including Covariate Data on BMI and Smoking

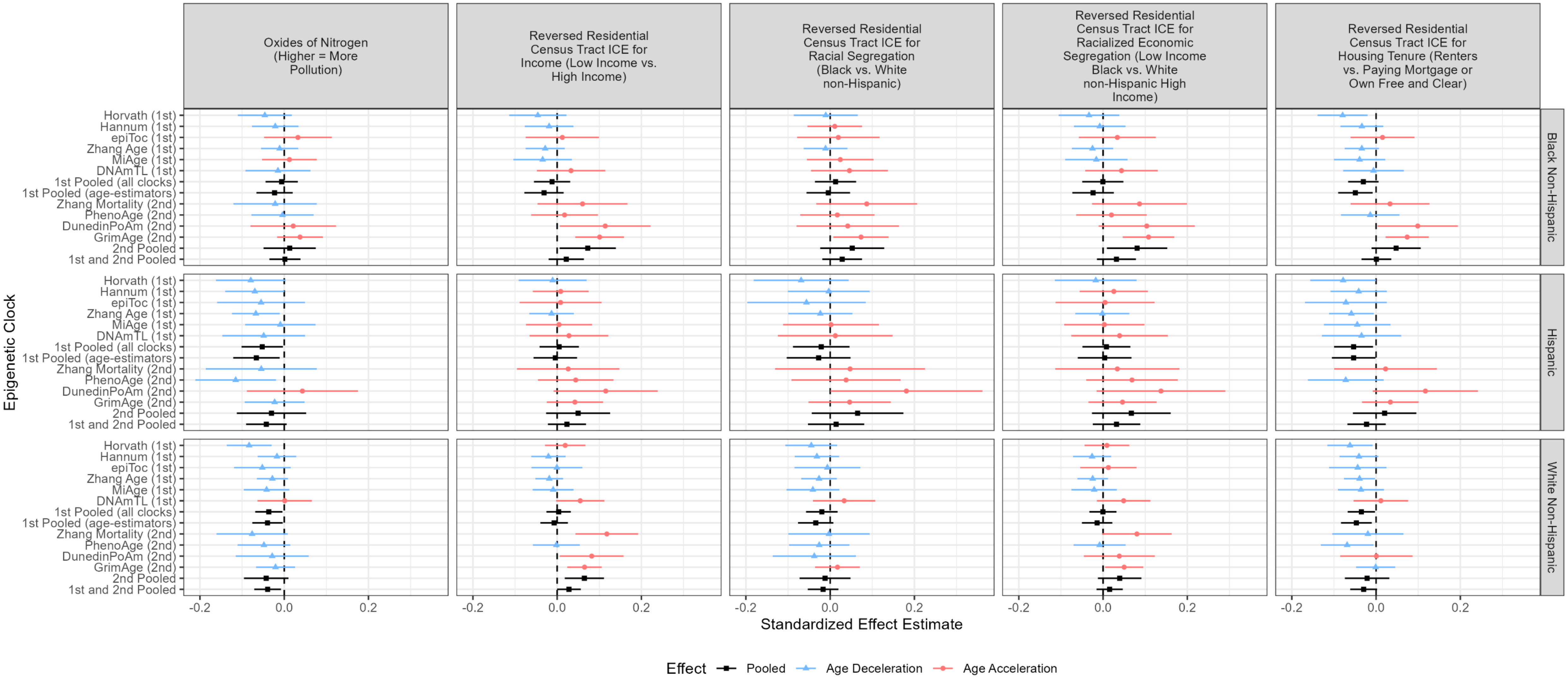

* unadjusted for multiple comparisons

© 2024 Krieger N et al. *JAMA Network Open.*
