# Supplementary material for: Epigenetic Aging and Racialized, Economic, and Environmental Injustice: NIMHD Social Epigenomics Program
Source: JAMA Netw Open. 2024 Jul 29;7(7):e2421832. doi: 10.1001/jamanetworkopen.2024.21832 (PMC11287398; doi:10.1001/jamanetworkopen.2024.21832)
Supplement: Supplement 2. — Data Sharing Statement [file jamanetwopen-e2421832-s002.pdf]

Data Sharing Statement

Krieger. Epigenetic Aging and Racialized, Economic, and Environmental Injustice. *JAMA Netw Open*. Published July 29, 2024. doi:10.1001/jamanetworkopen.2024.21832

Data
Data available: Yes
Data types: Deidentified participant data, Data (not involving human participants), Data dictionary
How to access data: See for all URLs: https://www.hsph.harvard.edu/nancy-krieger/data-sharing-resources/ • ICE metrics relating to racial composition, income distribution, and housing tenure that were derived from sources in the public domain i.e. the US Census and the American Community Survey are available at the census tract level now on GitHub (https://github.com/DNAmAndAdversity/CensusData) • The State Policy Liberalism Index data used in our study is also publicly available and can be obtained from the Harvard Dataverse. Reference: Caughey, Devin; Warshaw, Christopher, 2014, "The Dynamics of State Policy Liberalism, 1936-2014", http://dx.doi.org/10.7910/DVN/ZXZMJB Dataverse [Distributor] V1 [Version]. • De-identified data from the My Body My Story study used for this project will be made available only for purposes approved by the study PI, as stipulated by the study's informed consent protocol. The application form to obtain these data will be made available via this website after completion of this project in late Fall 2024. • Data from the Multi-Ethnic Study of Atherosclerosis (MESA) must be obtained directly from the MESA website via their application protocol (https://internal.mesa-nhlbi.org/) • The scripts to create the epigenetic clocks constructed from raw methylation data are available on GitHub (https://github.com/shwatkins/epigenetic_clocks_mbms_mesa)
When available: beginning date: 12-31-2024

Supporting Documents
Document types: Statistical/analytic code
How to access documents: See for all URLs: https://www.hsph.harvard.edu/nancy-krieger/data-sharing-resources/ • Code used to construct the variables as well as code used for our analyses are also available on GitHub (https://github.com/DNAmAndAdversity/DNAmAndAdversity_public) and here (https://doi.org/10.5281/zenodo.10256923)
When available: With publication

Additional Information
Who can access the data: Publicly available data are available to anyone, directly accessible from our website. Restricted data can be used by researchers whose proposed use of the data is approved, separately for the My Body My Story Study and for the MESA Study.
Types of analyses: Publicly available data are available to anyone, directly accessible from our website. Restricted data can be used only for a specified purpose in accord with the mission of and scientific approval of each study (My Body My Story and MESA).
Mechanisms of data availability: No investigator support is provided for use of the data. The publicly accessible data are available without approval. The restricted use data are available only after approval by the relevant study (My Body My Story and MESA).
Any additional restrictions: Use of My Body My Story and MESA data must be in compliance with all human subjects data requirements / IRB approval requirements of each study.
